# Nanotherapy for Neural Retinal Regeneration

**DOI:** 10.1002/advs.202409854

**Published:** 2025-01-14

**Authors:** Chuyao Yu, Li Dong, Yujia Lv, Xuhan Shi, Ruiheng Zhang, Wenda Zhou, Haotian Wu, Heyan Li, Yitong Li, Zhou Li, Dan Luo, Wen‐Bin Wei

**Affiliations:** ^1^ Beijing Tongren Eye Center Beijing Key Laboratory of Intraocular Tumor Diagnosis and Treatment Beijing Ophthalmology&Visual Sciences Key Lab Medical Artificial Intelligence Research and Verification Key Laboratory of the Ministry of Industry and Information Technology, Beijing Key Laboratory of Intelligent Diagnosis, Treatment and Prevention of Blinding Eye Diseases Beijing Tongren Hospital Capital Medical University Beijing 100730 China; ^2^ Beijing Institute of Nanoenergy and Nanosystems Chinese Academy of Sciences Beijing 101400 China

**Keywords:** retinal regeneration, retinal disease, nanotechnology, nanoparticles

## Abstract

Retinal diseases can severely impair vision and even lead to blindness, posing significant threats to both physical and mental health. Physical retinal regenerative therapies are poised to revolutionize the treatment of various disorders associated with blindness. However, these therapies must overcome the challenges posed by the protective inner and outer blood‒retinal barriers. Nanotechnology applications in ophthalmology have shown great potential in addressing the issue of drug delivery to the eye. Moreover, nanotechnology‐based therapeutics can have profound clinical impacts on retinopathy, particularly retinal regeneration, thereby improving patient outcomes. Continuous advancements in nanotechnology are being applied to regenerate lost or damaged eye tissues and to treat vision loss and blindness caused by various retinal degenerative diseases. These approaches can be categorized into three main strategies: i) nanoparticles for delivering drugs, genes, and other essential substances; ii) nanoscaffolds for providing biocompatible support; and iii) nanocomposites for enhancing the functionality of primary or stem cells. The aim of this comprehensive review is to present the current understanding of nanotechnology‐based therapeutics for retinal regeneration, with a focus on the perspective functions of nanomaterials.

## Graphical abstract

Nanotechnology enhances ophthalmic treatments by improving drug delivery and regenerating ocular tissues, combating vision loss from retinal diseases through innovative nano‐systems. This review outlines ocular anatomy, pathology, immune microenvironment, and barriers, detailing nanocarrier characteristics, classification, and preparation methods, and listing typical nanotechnology‐based treatment cases. It enriches understanding of nanotechnology‐driven retinal regeneration therapy.

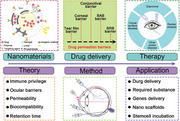

## 1 Introduction

Many retinal diseases cause severe vision impairment and can even lead to blindness. Visual disorders are generally categorized into optical blindness and neural blindness. While optical blindness, caused by conditions such as cataracts and keratopathy, can often be effectively treated with surgery, neural blindness is more challenging to address due to nerve damage. Unfortunately, current methods are inadequate for preventing the progression of such diseases. Retinal diseases pose a significant challenge in ophthalmology, affecting millions of people worldwide and leading to vision loss or total blindness.^[^ ^1^ ^]^ Diseases such as age‐related macular degeneration, diabetic retinopathy, and retinitis pigmentosa deteriorate retinal photoreceptor cells, resulting in irreversible vision loss. Given the serious consequences of these diseases, an urgent and ongoing need is effective therapeutics. Presently, the main treatment options for retinal diseases include pharmacological, surgical, and regenerative therapies. Among these, physical retinal regenerative therapies, which aim to restore or replace damaged ocular tissues, are emerging as revolutionary alternatives. However, these therapies face challenges in safely and efficiently delivering reparative factors across the inner and outer blood‒retinal barriers, which protect the ocular tissue from the systemic circulation.

Unlike other organs, the eye's immune‐privileged status means that it does not mount an immune response against tissue grafts. While this characteristic can be advantageous for the introduction of new substances into the eye, it also limits the ability of the eye to regenerate tissues effectively.^[^ ^2^ ^]^ Therefore, extensive efforts have been made over the past decades to regenerate lost or damaged eye tissues to treat vision loss caused by retinal degenerative diseases, trauma, infections,^[^ ^3^ ^]^ and inherent genetic retinal defects.^[^ ^4^ ^]^ Early‐ and middle‐stage retinal neuronal damage can be partially repaired by drugs,^[^ ^5^ ^]^ but most patients present with late‐stage disease. At this stage, only a few functional nerve cells remain in the retina, and DNA repair approaches and drugs are ineffective at reversing neurological blindness.^[^ ^6^ ^]^ Moreover, the retina has a minimal regenerative capacity, making replacement therapy essential for protecting and regenerating the retinal optic nerve in patients with advanced retinal neuronal damage.^[^ ^7^ ^]^ Traditional replacement therapies involve substituting damaged visual tissue with artificial visual prostheses,^[^ ^8^, ^9^ ^]^ which receive optical signals via microelectrodes implanted in different areas of the eye.^[^ ^10^ ^]^ However, artificial retina prostheses cannot fully simulate the biological functions of the retina due to the limited number of electrodes, poor biocompatibility, and opacity.^[^ ^9^, ^11^ ^]^ Another traditional approach, nontargeted retinal stem cell transplantation, involves injecting stem cells or stem cell‐derived retinal neurons via the subretinal and intravitreal routes.^[^ ^12^ ^]^ However, these therapies are far from clinical application due to complications such as inflammatory responses and retinal detachment.

**Figure 1:**
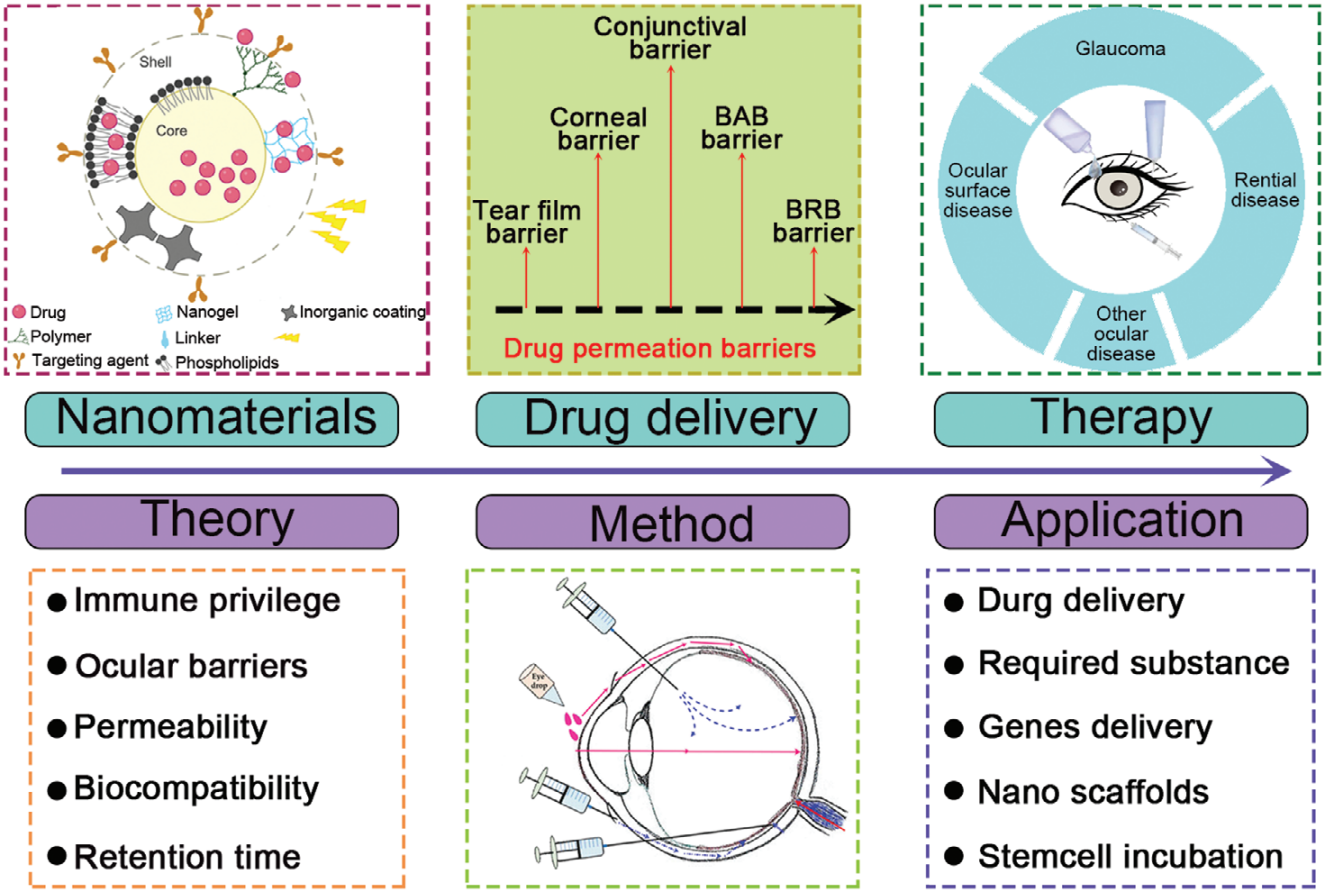
An overview of neural retinal regeneration based on nanotherapy: theory, method, and application. Reproduced with permission.^[^ ^194^ ^]^ Copyright 2022, Frontiers. Reproduced with permission.^[^ ^195^ ^]^ Copyright 2020, The Royal Society of Chemistry.

Nanotechnology, which involves structures and systems at the nanometer scale, is emerging as a promising vehicle for targeted regenerative therapies across various medical fields, including ophthalmology.^[^ ^13^, ^14^, ^15^ ^]^ Compared with most traditional drugs, nanomaterials offer advantages such as a smaller size, ease of preparation, lower degree of irritation to biological tissues, and biodegradability. Different nanomaterials may be suitable for carrying drugs with varying chemical characteristics.^[^ ^16^ ^]^ Common materials used in nanocontrolled release systems include nanoparticles, nanowires, and modified or coated hybrid nanomaterials.^[^ ^17^ ^]^ Nanoparticles have been applied in the treatment of retinal degenerative diseases.^[^ ^18^ ^]^ This review explores the theory and applications of nanotherapy in neural retinal regeneration (**Figure** **1**) from the following perspectives: 1) the mechanism of nanotherapy in neural retinal regeneration; 2) the characterization, preparation, and advantages of nanomaterials in nanotherapy; and 3) nanotherapy‐based vision restoration strategies.

## 2 Mechanism of Nanotherapy in Neural Retinal Regeneration

### 2.1 The Anatomical Structure of the Eye and its Pathological Mechanisms

**Figure 2:**
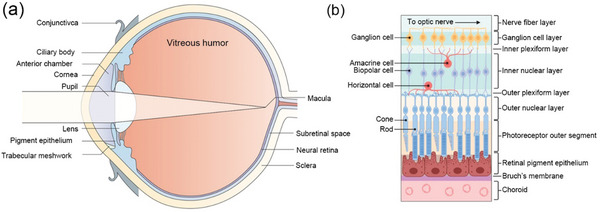
Anatomy of the visual axis of the eye. a) Initially, the light image enters through the cornea and passes through the anterior chamber, pupil, lens, and vitreous body, eventually reaching the photoreceptor cells, which form the outermost layer of the neural retina. Reproduced with permission.^[^ ^196^ ^]^ Copyright 2003, Springer Nature Limited. b) The structure of the retina and its layers seamlessly transform light into perceivable images, coordinating precise visual functions. Reproduced with permission.^[^ ^19^ ^]^ Copyright 2021, Frontiers.

The complex structure of the human eye can be divided into three layers (**Figure** **2**a), each containing various components, such as the aqueous humor, lens, and vitreous body. The dimensions of the eye differ between individuals by only 1–2 mm, corresponding to a consistent weight of 7.5 g and a volume of 6.5 cm^3^. The outermost membrane consists of the cornea and sclera. The intermediate layer contains the major blood vessels that supply the eye, beginning with the choroid at the back, passing through the ciliary body, and extending to the iris. The innermost retinal layer lies on top of the choroid, receiving most of its nourishment from the choroid's vessels, with the remaining supply supplemented by the retinal vessels visible through an ophthalmoscope. The ciliary body and iris are covered by a very thin layer comprising the ciliary epithelium and the posterior epithelium of the iris, both of which are interconnected with the retina. The crystalline lens lies within the cavities formed by these three layers, and it is delicately suspended from the ciliary body by transparent fibers known as the suspensory ligament or Zinn's zone. Other components include the aqueous humor, a transparent fluid that fills the space between the cornea, lens, and iris, and the vitreous body, a transparent jelly that occupies the larger cavity surrounded by the sclera, ciliary body, and lens. The anterior chamber of the eye is the space between the cornea and the front surfaces of the iris and lens, whereas the posterior chamber is a smaller space between the rear surface of the iris and the ciliary body, suspensory ligament, and lens. Both chambers contain aqueous humor and communicate through the pupil.

The retina, an extension of the forebrain, plays a crucial role in converting light into chemical energy, triggering neural activity that communicates with the brain. Generally, the retina consists of four main layers^[^ ^19^ ^]^ (Figure 2b). 1) The pigment epithelium is situated adjacent to the choroid, as previously mentioned. 2) Above the epithelium is a layer containing rods and cones, which are light‐sensitive cells. Changes in these cells induced by light are transmitted to 3) a layer of nerve cells known as bipolar cells. These bipolar cells connect with 4) the innermost layer of nerve cells, ganglion cells, which transmit signals from the eye along their projections, or axons, forming optic nerve fibers. Therefore, the optic nerve functions more like a central tract than a typical nerve, connecting two areas of the nervous system: the bipolar cell layer and the cells of the lateral geniculate body, a visual relay station located at the rear of the forebrain. The organized arrangement of retinal cells creates the outer nuclear layer, which contains the nuclei of the rods and cones; the inner nuclear layer, which houses the nuclei and perikaryon (the main cell bodies outside the nucleus) of the bipolar cells; and the ganglion cell layer, which contains the structures of the ganglion cells. Plexiform layers are regions where neurons form connections. The outer plexiform layer contains projections from rods and cones, which terminate as rod spherules and cone pedicles, forming connections with the dendritic processes of bipolar cells. These connections allow changes induced in the rods and cones by light to be transmitted to the bipolar cells. The axons of the bipolar cells and the dendritic processes of the ganglion and amacrine cells are located in the inner plexiform layer. These connections enable messages in bipolar cells to be transmitted to ganglion cells, which then send signals outward as optic nerve messages along ganglion cell axons.

Retinal degeneration is a common manifestation of various ocular diseases, including DR, AMD, and pigmentary retinal disorders. In DR, damage to and cell death of capillary endothelial cells lead to retinal oxygen deprivation. High blood glucose levels can also directly damage neurons and other cells within the eye. AMD and retinitis pigmentosa are typically caused by the death or dysfunction of light‐sensitive retinal cells. Cell death can be triggered by factors such as oxidative stress, inflammation, and autoimmune responses at the cellular level. Expanding our understanding of the mechanisms involved in retinal degeneration may aid in the development of new therapeutic strategies. These effects could include protecting retinal cells from slow degeneration or stimulating the regeneration of retinal cells to repair the damaged retina.

### 2.2 Ocular Specialty of Immune Privilege

Immune privilege within the eye relies on various molecular and biochemical interactions, as well as specific ocular immune features, such as the limited lymphatic drainage of internal structures. The continuous flow of aqueous humor also helps expel antigens from the eyes into the bloodstream. This immune privilege protects the eye from intraocular inflammation. The known immunosuppressive mechanisms include a specialized microenvironment within the eye, consisting of ocular fluids, the blood‒retina barrier, and resident ocular parenchymal cells.^[^ ^20^ ^]^ The molecular mechanisms underlying the development and maintenance of ocular immune privilege involve regulatory T cells (Tregs) generated by anterior chamber‐associated immune deviation (ACAID) and ocular resident cells, including corneal endothelial cells, ocular pigment epithelial cells, and the aqueous humor.^[^ ^20^ ^]^

#### 2.2.1 Immune Privilege Derived from Eye‐Derived Antigens

**Figure 3:**
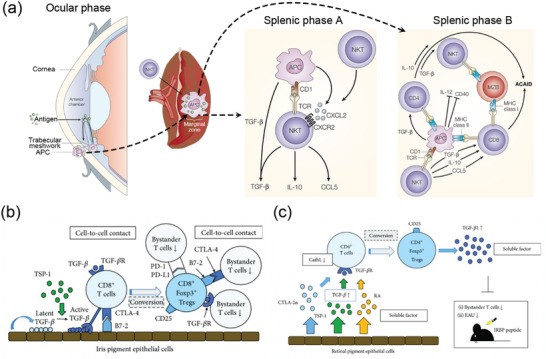
Schematic diagram of the mechanism of origin of ocular immunity. a) Immune privilege derived from eye‐derived antigens can be divided into three phases: the ocular phase, splenic phase A, and splenic phase B. Reproduced with permission.^[^ ^196^ ^]^ Copyright 2003, Springer Nature Limited. b) Molecular mechanism underlying the generation of regulatory Tregs by murine iris pigment epithelial (PE) cells. Reproduced with permission.^[^ ^20^ ^]^ Copyright 2018, Wiley. c) Molecular mechanism underlying the generation of regulatory Tregs by murine RPE cells. Reproduced with permission.^[^ ^20^ ^]^ Copyright 2018, Wiley.

The eye employs various strategies to maintain immune privilege and prevent inflammation, which could lead to vision loss.^[^ ^21^ ^]^ One such strategy is the induction of peripheral tolerance known as ACAID (**Figure** **3**a).^[^ ^22^ ^]^ During ACAID, antigens in the eye's anterior chamber elicit a systemic immune response that promotes the retention of primed cytotoxic T‐cell precursors and B cells producing noncomplement‐fixing IgG1 antibodies. Simultaneously, ACAID inhibits CD4^+^ type 1 T helper (Th1) and type 2 T helper (Th2) cells, as well as B cells, which produce complement‐fixing antibodies.^[^ ^23^ ^]^ Research has shown that the spleens of mice exposed to antigens in the anterior chamber develop three types of antigen‐specific regulatory traits that mediate ACAID.^[^ ^24^ ^]^ CD4^+^ Tregs, known as “afferent regulators”, suppress the initial activation and differentiation of naive Tregs into Th1 effector cells. CD8^+^ Tregs, referred to as “efferent regulators”, inhibit the expression of Th1 immune responses and the switching of B cells to the complement‐fixing IgG isotype. Efferent CD8^+^ Tregs act in the periphery, including the eye, whereas afferent CD4^+^ Tregs act in secondary lymphoid organs. In ACAID, eye‐derived antigen‐presenting cells (APCs) induce the expansion of tolerogenic B cells, which in turn contribute to the production of antigen‐specific Tregs. Invariant natural killer Tregs also play a role in the generation of ACAID. Additionally, experiments have shown that the injection of certain retinal antigens into the anterior chamber can impair the development of delayed hypersensitivity reactions and prevent experimental autoimmune uveoretinitis (EAU), a model of human uveitis. The adoptive transfer of splenocytes from mice exposed to retinal antigens in the anterior chamber has been shown to suppress ongoing intraocular inflammation, indicating a role for ACAID‐induced Tregs in suppressing the immune response. Recent research has also highlighted the role of retinal antigen‐pulsed tolerogenic APCs in suppressing EAU through the induction of CD8^+^ Tregs, which can suppress the activity of Tregs (including Th1 and Th17 cells).^[^ ^25^ ^]^ In summary, ACAID is an immune tolerance mechanism induced by the eye to prevent sight‐destroying inflammation. This process involves the expansion of antigen‐specific Tregs and the inhibition of certain immune cell activities, ultimately suppressing autoimmune reactions in the eye.

#### 2.2.2 Immune Privilege Derived from the Intraocular Microenvironment

An increasing body of evidence points to key roles of ocular resident cells, including corneal endothelial (CE) cells, pigment epithelial (PE) cells, and retinal pigment epithelial (RPE) cells, in establishing and preserving an immunosuppressive intraocular environment. This activity is facilitated by the induction of Tregs (CD4^+^ and CD8^+^),^[^ ^26^ ^]^ which are crucial for ensuring immune tolerance and homeostasis by inhibiting the damage caused by overactive immune responses.^[^ ^27^ ^]^ This section explores the molecular mechanisms by which ocular resident cells induce Tregs and examines the potential benefits of CE‐, PE‐, and RPE‐induced Tregs in maintaining ocular immune privilege.

CE cells, located on the inner surface of the anterior chamber and in contact with the aqueous humor, also promote local immune tolerance in the human eye. Activated Tregs exposed to CE cells are unable to develop effector Treg functions.^[^ ^28^ ^]^ Additionally, studies have shown that mouse CE cells consistently express Fas ligand, programmed death ligand 1 (PD‐L1/CD274), glucocorticoid‐induced tumor necrosis factor receptor family‐related protein ligands, and other immunoregulatory molecules, which induce the apoptosis of effector Tregs.^[^ ^29^ ^]^

Ocular PE cells, which are found in the iris, ciliary body, and retina, play critical roles in establishing and maintaining ocular immune privilege (Figure 3b).^[^ ^21^, ^30^ ^]^ Iris PE cells have been shown to inhibit the anti‐CD3‐driven activation of primed or naive Tregs.^[^ ^30^ ^]^ Studies have indicated that iris PE cells prevent TCR‐driven T‐cell activation in vitro through direct cell contact, where B7‐2 (CD86) expressed by iris PE cells interacts with CTLA‐4 on responsive Tregs.^[^ ^31^ ^]^ B7‐2^+^ iris PE cells promote the selective activation of CTLA‐4^+^ CD8^+^ Tregs in the presence of anti‐CD3 agonistic antibodies and secrete high levels of active TGF‐β, resulting in the overall inhibition of the total T‐cell population, including CD4^+^ Tregs.^[^ ^32^ ^]^ Additionally, studies have revealed that the subretinal space can also be an immune‐privileged site, with RPE cells serving as immune‐privileged tissues. RPE cells are instrumental in preserving immune privilege in this subretinal space.

RPE cells secrete soluble factors such as TGF‐β, TSP‐1, and PGE2, which serve as mediators that modulate innate and adaptive immune responses (Figure 3c). In a manner dependent on inflammatory conditions, RPE cells can inhibit activated T cells through the regulated expression of MHC class II molecules.^[^ ^33^ ^]^ Furthermore, in the presence of inflammatory cytokines such as IL‐17 and IFN‐γ, RPE cells express high levels of PD‐L1, which can suppress the pathogenic activity of IRBP‐specific T cells that induce EAU.^[^ ^34^ ^]^

### 2.3 Ocular Barriers and Drug Delivery Systems

#### 2.3.1 Ocular Barriers

##### Tear Film Barrier

**Figure 4:**
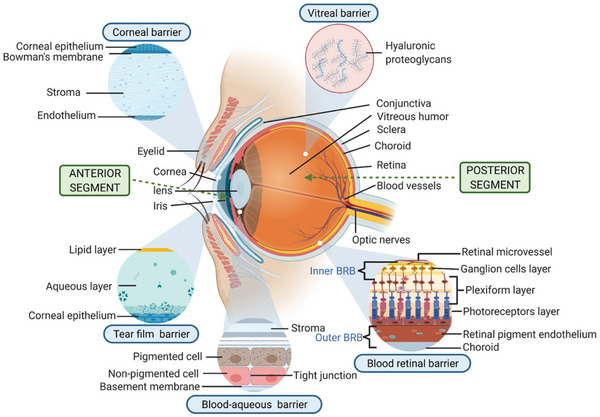
Physiological barriers in ocular drug delivery. Reproduced with permission.^[^ ^197^ ^]^ Copyright 2022, Springer Nature.

The drainage of administered eye solutions represents a significant challenge in the precorneal area (**Figure** **4**). The drug can be lost through the tear fluid due to tear production, drainage of the solution, and inefficient absorption in the conjunctiva.^[^ ^35^ ^]^ Furthermore, drug metabolism and protein binding can further hinder drug absorption. The constant renewal of tear fluid helps maintain eye hydration and prevents dust or pathogens from accumulating on the eye. The duration of the administered formula must be extended to ensure that the drug remains effective, a goal that can be achieved through various mechanisms.

##### Corneal Barrier

The cornea serves as a robust barrier against various types of chemical and mechanical damage while assisting in focusing light onto the retina. The cornea consists of three layers: the epithelium, stroma, and endothelium. The epithelium is composed of five to seven layers of tightly interconnected cells. The stroma is a dense layer filled with water. As a barrier, the epithelium impedes the passage of hydrophilic drugs and larger molecules, whereas the stroma obstructs lipophilic drugs.^[^ ^36^ ^]^ The endothelium maintains the clarity of the cornea and regulates the entry of hydrophilic drugs and macromolecules into the aqueous humor. Generally, the diffusion of drugs through the cornea is influenced by the molecular weight, charge, degree of ionization, and hydrophobicity of the drug. Therefore, transcorneal permeation is considered the rate‐limiting step in the drug's transition from the tear fluid to the aqueous humor.

##### Conjunctival Barrier

The conjunctival barrier plays a crucial role in the eye's protective apparatus; it acts as an immunoreactive tissue enveloping the rear section of the eyelids and the anterior portion of the sclera, significantly contributing to eye health. Owing to its unique structure, abundant blood vessels, and variety of inherent immune cells, the conjunctiva serves as a critical barrier against environmental disturbances, foreign entities, and disease‐causing agents. The epithelial cells of the conjunctiva form the primary line of defense by establishing physical and biochemical barriers against pathogens. These barriers include tight junction proteins that seal the spaces between cells, thereby inhibiting pathogen invasion. Additionally, the conjunctiva plays an essential role in tear production and lubrication of the ocular surface, assisting in the elimination of microorganisms and debris.

##### Vitreal Barrier

The vitreous humor is a considerable obstacle to the infiltration of nanoparticulate‐based systems delivering ocular drugs. Both its gel‐like structure and biochemical composition can influence the rate at which nanoparticles traverse the tissue to reach the retinal area. Factors such as the configuration of the vitreous, internal flow mechanisms, age‐related changes, and the prevalence of inflammation can also affect this movement. The physical characteristics of the nanoparticle formulation are crucial when designing a system that can permeate the vitreous barrier and effectively target the posterior part of the retina.^[^ ^37^ ^]^

##### Blood–Ocular Barriers

These barriers function as gatekeepers, preventing foreign substances from entering the bloodstream. They are divided into the blood‒aqueous barrier (BAB) and the blood‒retinal barrier (BRB). The BAB, located in the anterior part of the eye, prevents many compounds from infiltrating the intraocular environment. Notably, the BAB allows the passage of lipophilic and small drugs, which are expelled from the anterior compartment more quickly than their hydrophilic and larger counterparts. For example, pilocarpine is cleared faster than inulin. Conversely, the BRB is located in the posterior part of the eye and is composed of retinal endothelial cells and retinal pigment epithelial cells. It serves to restrict the entry of potentially harmful substances, water, and plasma components into the retina.^[^ ^38^ ^]^

#### 2.3.2 Drug Delivery Systems

**Figure 5:**
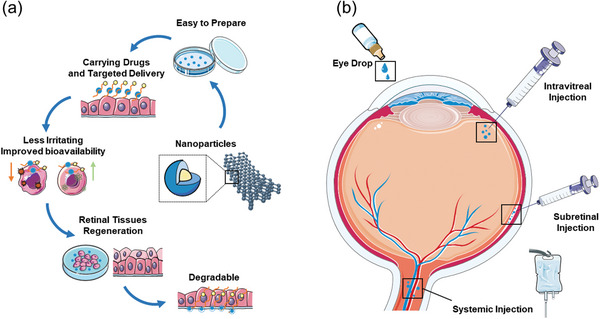
Nanoparticles delivered via the ocular drug delivery system promote retinal regeneration. a) Nanoparticles, prepared for carrying drugs and targeted delivery, can reduce irritation and improve bioavailability to promote retinal tissue regeneration due to their intrinsic advantages. b) Both topical applications (shown at the top, e.g., eye drops) and invasive applications (shown on the left, e.g., intravitreal, sub‐retinal, and systemic injections) are commonly used for the ocular delivery of nanoparticles.

Previous studies have shown that the use of nanomaterials as drug carriers can increase the bioavailability of drugs, reduce their side effects, prolong drug retention in local tissues, decrease both the required dosage and frequency of administration, and clearly improve multiple aspects of drug delivery.^[^ ^39^ ^]^ As illustrated in **Figure** **5**a, various nanovesicles have been employed for specific drugs, genes, and necessary substances.^[^ ^40^ ^]^ These nanovesicles can be coated with ligands to target specific cell surface receptors, with polymers to extend their half‐life in the circulatory system,^[^ ^41^ ^]^ and with specific materials that enable them to penetrate the entire retina.^[^ ^42^ ^]^ This design effectively overcomes the challenges presented by the blood‒retinal barrier for drug, gene, and necessary substance delivery. When designing a nanoparticle system, particular attention must be given to both the preparation of the vesicles or nanoparticles and their delivery.

Several methods are available for preparing polymeric vesicles, including the solvent switch method, film rehydration, solid rehydration (bulk rehydration), and electroformation.^[^ ^43^ ^]^ When designing polymeric vesicles, biocompatible materials must be considered to ensure their effectiveness and stability. For example, poly(ethylene glycol) (PEG) is widely recognized for enhancing drug solubility and stability in plasma while reducing immunogenicity when it is integrated with different molecules.^[^ ^44^ ^]^ Coating nanoparticles, including liposomes, with albumin and/or PEG can create a hydrophilic surface that temporarily resists protein adsorption, thereby prolonging particle bioavailability.^[^ ^44^ ^]^

Choosing an appropriate method for nanoparticle delivery is equally important for improving treatment outcomes (Figure 5b). The methods used to reach the retina fall into two categories: topical (eye drops) and invasive (systemic, intravitreal, and subretinal injections^[^ ^4^, ^45^ ^]^). When selecting a method, solubility, sorption, mobility, bioavailability, and associated risks must be considered. Currently, intravitreal and subretinal injections are regarded as the most effective and common methods for delivering drugs or genes to retinal ganglion cells (RGCs^[^ ^46^ ^]^). Intravitreal injections are typically used for targeting RGCs and inner retinal interneurons,^[^ ^47^ ^]^ whereas subretinal injections are more effective at reaching the outer retina.^[^ ^48^ ^]^ The ideal route of administration should ensure that the nanoparticle remains functional upon reaching the target cell and that the optimal nanoparticle concentration is achieved in the target tissue with minimal side effects to facilitate retinal regeneration.

## 3 Characterization, Preparation, and Advantages of Nanomaterials in Nanotherapy

### 3.1 Characteristics of Ocular Nanotherapy Nanocarriers

Nanotechnology involves working with structures at the nanoscale level, typically ranging from 1 to 100 nm, which is proportionally similar to peptide drugs.^[^ ^49^ ^]^ Key physicochemical properties, such as permeability, biocompatibility, drug loading, retention time, and stability, are intricately linked to their therapeutic effectiveness in treating ocular pathologies.^[^ ^50^ ^]^ By characterizing these physicochemical and biological attributes of nanocarriers, we can obtain valuable insights to improve the design of efficient novel delivery systems.

#### 3.1.1 Permeability

**Figure 6:**
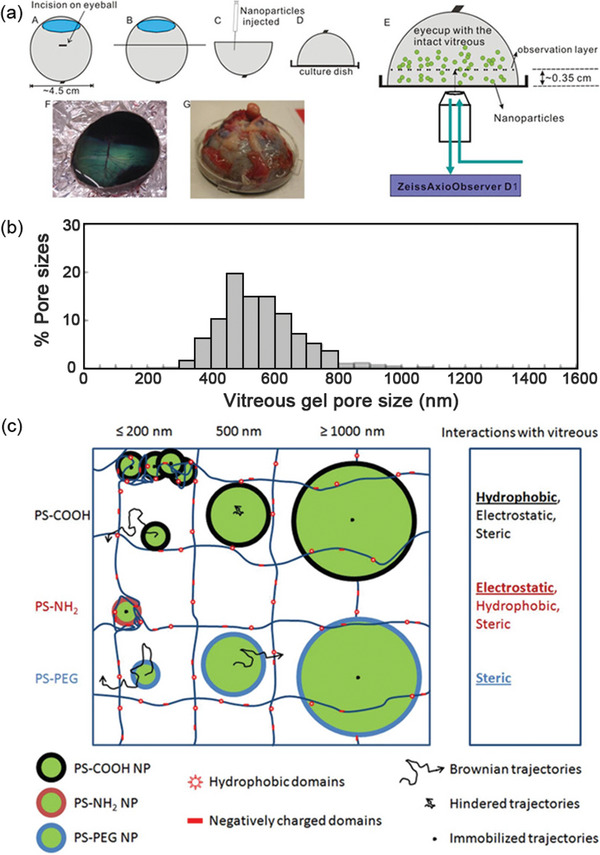
a) Schematic depicting set‐up for multiple particle tracking in fresh, intact central bovine vitreous. Reproduced with permission.^[^ ^52^ ^]^ Copyright 2013, Elsevier B.V. b) Distribution of the effective pore size of the central bovine vitreous was estimated by obstruction scaling model, with average pore size 550 ± 50 nm. Reproduced with permission.^[^ ^52^ ^]^ Copyright 2013, Elsevier B.V. c) Schematic illustrating effect of size and surface chemistry on nanoparticle transport in the vitreous meshwork. Hydrophobic, electrostatic and steric effects all contributed to the particle transport in vitreous meshwork. Reproduced with permission.^[^ ^52^ ^]^ Copyright 2013, Elsevier B.V.

Charge and particle size are crucial factors influencing the permeability of nanoparticles. The vitreous is a network tissue with a pore size of ≈550 nm (**Figure** **6**a,b) and is composed of ≈1% biomass (collagen and hyaluronan) and 99% water.^[^ ^51^ ^]^ The vitreous carries a negative charge due to hyaluronan, which facilitates the passage of negatively charged particles smaller than 550 nm through the vitreous barrier. As illustrated inFigure 6c, 510 nm negatively charged particles coated with polyethylene glycol can easily penetrate the vitreous, whereas 100–200 nm positively charged particles coated with primary amine groups become trapped within the vitreous network. Furthermore, the transport of large nanoparticles (>1000 nm) in the vitreous is limited, regardless of the surface chemical modification.^[^ ^52^ ^]^ After passing through the vitreous, nanoparticles must cross the inner limiting membrane (ILM), outer limiting membrane (OLM), and Müller cells.^[^ ^53^ ^]^ The ILM is also negatively charged, which affects nanoparticle transport. Although ILMs and OLMs freely allow the diffusion of small‐molecule drugs (10–20 nm), they still impede the delivery of macromolecules, biologics, and nanoparticles. Studies suggest that most positively charged nanoparticles cannot overcome the ILM, whereas neutral or negatively charged nanoparticles can. However, some findings contradict these results, showing that negatively charged nanoparticles can pass through the ILM despite their larger size. For example, 325 nm negatively charged nanoparticles were found to be transported across the ILM in rats.^[^ ^54^ ^]^

Permeability also strongly depends on the nanoparticle shape. In general, spherical nanoparticles exhibit lower penetration than other shapes do,^[^ ^55^ ^]^ which should be considered when designing clinical nanomedicines. Several studies on nanotherapy have explored the impact of the nanoparticle shape on permeability. For example, Jain and colleagues demonstrated that 1 h after injection in mice, nanorods infiltrated 1.7 times more effectively than nanospheres of the same hydrodynamic diameter (33–35 nm).^[^ ^56^ ^]^ Additionally, an intravenous injection of AuNPs in the form of nanocages, nanospheres, and nanodiscs into mice revealed that the nanocages penetrated the core of the target site, whereas the nanospheres and nanodiscs remained at the periphery.^[^ ^57^ ^]^ Rod‐shaped nanoparticles with aspect ratios of 3.5, 7, and 16.5 were injected into mice, and those with an aspect ratio of 7 exhibited the best permeability, indicating that the aspect ratio significantly influences nanoparticle permeability.^[^ ^58^ ^]^

#### 3.1.2 Biocompatibility

**Figure 7:**
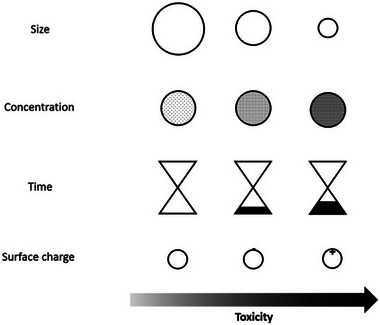
Factors affecting toxicity of nanoparticles on neuronal cells. Image reprinted and adapted with permission from multidisciplinary digital publishing institute (MDPI). Reproduced with permission.^[^ ^198^ ^]^ Copyright 2017, Elsevier Inc. All rights reserved.

When treating retinal diseases with nanotherapy, the toxicity of nanoparticles to neuronal cells must be considered. Factors such as the nanoparticle size, dose, administration time, chemical composition, surface charge, and hydrophilicity are key determinants of neuronal toxicity (**Figure** **7**).^[^ ^59^ ^]^ The mechanisms of neuronal toxicity include the removal of reactive oxygen species (ROS) and changes in gene expression patterns.^[^ ^60^ ^]^ Additionally, microglia can phagocytize nanoparticles, leading to the generation of intracellular ROS and reactive nitrogen species,^[^ ^61^ ^]^ as well as the expression of nitric oxide synthase and the secretion of TNF, IL‐1β, and IL‐6.^[^ ^62^ ^]^

Potential adverse effects on the tolerance of the eyes may occur despite the evaluation of the toxicity and safety of nanoparticles. Research has shown that the accumulation of certain nanomaterials in eye tissue may activate the immune response in the eyes, leading to allergic reactions,^[^ ^63^ ^]^ chronic inflammation and cellular damage. Although nanoparticles are generally considered as anti‐inflammatory components, certain nanomaterials may also generate free radicals, causing oxidative stress, damaging cell membranes, proteins, and DNA, leading to cellular dysfunction or death.

Draize test (primary eye irritation test) was commonly applied to assess the side effect of nanomaterials in vivo,^[^ ^64^ ^]^ and HET‐CAM (hen's egg–chorioallantoic membrane) test was developed to assess the in vitro ocular tolerance.^[^ ^65^ ^]^ 3‐(4,5Dimethylthuazol‐2‐yl)‐2,5‐diphenyltetrazolium bromide (MTT) and 2′,7′‐dichlorodihydrofluorescein diacetate (H2DCFDA) assay was used to ensure that the synthesised nanoparticles were used to test cytotoxicity as well.^[^ ^66^ ^]^ In animal trials, eyeball tissues under nanoparticles‐injected could be evaluated including the presence of inflammatory eosinophils, neutrophils, mast cells or lymphocytes, and any other abnormality.^[^ ^67^ ^]^

Accordingly, the treatment of neural retinal degeneration with nanomaterials remains challenging, including how to effectively reduce the incidence of adverse reactions, to ensure the safety of treatment, and to optimize the design of nanomaterials to improve their biocompatibility and therapeutic efficacy.

#### 3.1.3 High Drug Load

Despite significant research efforts and substantial investments over the past decades, only a few nanomedicines have been approved by the U.S. Food and Drug Administration (FDA) for clinical use, such as Doxil (doxorubicin encapsulated by PEG‐coated liposomes) and Abraxane (albumin‐bound paclitaxel nanoparticles). One of the challenges hindering the clinical translation of nanomedicine is drug loading. Insufficient drug loading, coupled with uncontrolled drug release, represent major obstacles. Most nanomedicines exhibit low drug loading (typically a few weight percent), making their clinical translation difficult due to high production costs, issues with scalable production with reproducible properties, and potential toxic side effects of the nanomaterials. Additionally, achieving the therapeutic window often requires very high particle concentrations, leading to a viscous solution that complicates intravenous injection. Therefore, increasing drug loading is critical to minimize these adverse effects. A minimal amount of nanoparticles with high drug loading is needed to achieve therapeutic levels, which can reduce the potential adverse effects from overdosed materials and decrease the manufacturing costs of nanomedicines. These advantages are particularly beneficial for well‐tolerated drugs, allowing for significant dose escalation. Moreover, the route of administration directly impacts the formulation of nanomedicines. For oral delivery, the mucus gel layer is a significant barrier for nanoparticles reaching the absorption membrane, with a cutoff size for mucus permeability of ≈100 nm. For systemic intravenous administration, nanoparticles ranging from 10 to 150 nm are considered optimal for the enhanced permeability and retention (EPR) effect, although this EPR effect is under intense debate. Additionally, smaller nanoparticles may penetrate deeper, potentially achieving a better antitumor effect.

#### 3.1.4 Retention Time

Ocular retention is a critical characteristic of ocular delivery systems, as it extends the drug action period, reduces the frequency of drug administration, and enhances drug bioavailability.^[^ ^49^ ^]^ Nanosystems, such as thin films and hydrogels, with expansive surface areas allow for increased diffusion and prolonged contact on the corneal surface, thereby improving ocular retention. Techniques such as γ‐scintigraphy, fluorescence imaging, texture analysis, and surface plasmon resonance spectroscopy are commonly used to measure the intraocular retention of nanoformulations.^[^ ^68^ ^]^

#### 3.1.5 Stability

Stability challenges, including Ostwald ripening, creaming, coalescence, flocculation, and precipitation, represent significant hurdles in the development of nanocarriers.^[^ ^69^ ^]^ Short‐term stability, heating‒cooling cycles, centrifuge tests, freeze‒thaw cycles, and high‐temperature storage are methods used to evaluate the stability of various nanosystems.^[^ ^70^ ^]^ An emerging strategy to increase biological stability involves PEGylation. When PEG, a hydrophilic nonionic polymer with high chain flexibility, is used to coat or combine with the nanocarrier surface, it can prevent macrophage clearance by reducing interactions with the surrounding environment, such as oxidants, enzymes, and other degrading agents. Furthermore, in vivo drug flux studies have shown that pegylated nanostructured lipid carriers transport nearly double the levels of ciprofloxacin to all ocular tissues compared with their nonpegylated counterparts, as measured 2 h after administration.^[^ ^71^ ^]^

### 3.2 Various Types of Nanocarriers

**Figure 8:**
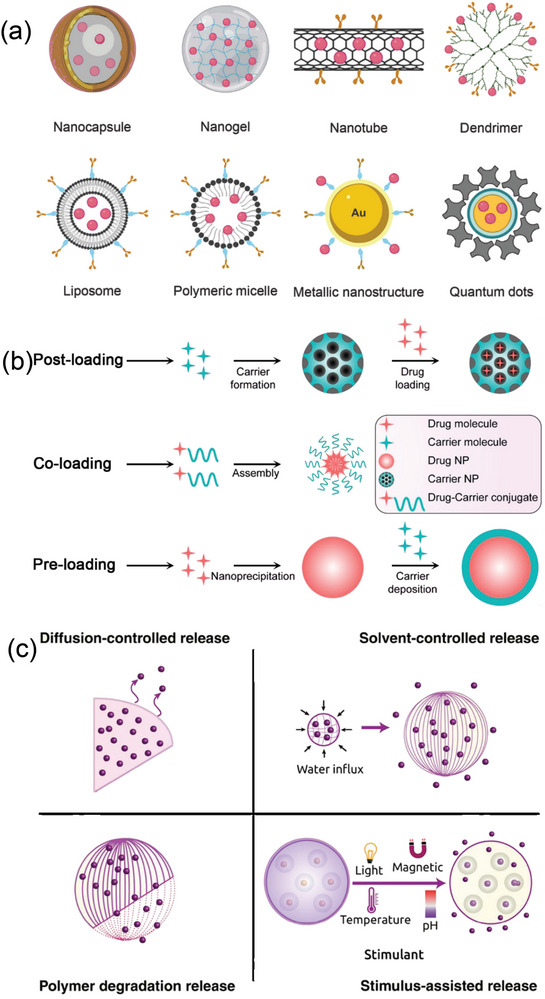
a) Schematic representation of different types of nanomaterials employed in ocular therapy, their important physical properties and surface chemistry required to carry drugs. Reproduced with permission.^[^ ^194^, ^199^ ^]^ Copyright 2022, Frontiers, Copyright 2019, Springer. b) Three representative strategies for making high drug‐loading nanoparticles. Reproduced with permission.^[^ ^200^ ^]^ Copyright 2020, Wiley‐VCH. c) Schematic depiction of diffusion‐, solvent‐controlled, polymer degradation, and other stimuli reliant drug release. Reproduced with permission.^[^ ^199^ ^]^ Copyright 2019, Springer.

This review discusses the development of “smart” nanomaterials for the treatment of visual disorders (**Figure** **8**a). We examine the design, fabrication, and application of various nanomaterials used in ocular therapy.

#### 3.2.1 Nanocapsules

Nanocapsules are nanoscale materials with a polymeric shell containing a liquid or solid core, making them ideal for targeted drug delivery systems.^[^ ^72^ ^]^ The polymeric shell protects the drug from degradation and can be functionalized for precise delivery. The choice of shell material significantly influences the stability, encapsulation efficacy, and other properties of nanocapsules, with biocompatible polymers such as PEG and PVA commonly used.^[^ ^73^ ^]^ Both natural and synthetic polymers can be utilized, and the process can be tailored to meet specific requirements. For example, polysaccharides such as chitosan offer desirable properties for nanocapsule synthesis, although their cationic charge can be a disadvantage and can be mitigated by the use of anionic polymers.^[^ ^74^ ^]^ Additionally, other polysaccharides and proteins have been used to create nanocapsules for various applications.^[^ ^75^ ^]^ Synthetic materials also provide advantages in terms of tunability, with the selection of materials largely depending on the specific application needs.

#### 3.2.2 Hydrogels

Hydrogels, 3D networks of hydrophilic polymer chains, have significantly advanced the treatment of ocular diseases due to their high water retention capacity and ability to transition from a liquid to a gel state upon contact with the eye.^[^ ^76^ ^]^ When initiated by heat, pH, or ion‐responsive materials, hydrogels improve and sustain drug delivery mechanisms by prolonging the retention time of drugs on the eye. They also allow the codelivery of multiple drugs. The combination of hydrogels and nanotechnology, such as nanoparticles, nanomicelles, microneedles, and nanofibers, increases the bioavailability of drugs. For example, a polypseudorotaxane hydrogel, an optimized hydrogel with shear‐thinning and sustained‐release properties, has shown improved drug retention and anti‐inflammatory effects on the treatment of anterior uveitis.^[^ ^77^ ^]^ Similarly, a hyaluronic acid hydrogel has shown promise for the long‐term treatment of wet AMD.^[^ ^78^ ^]^ Additionally, an injectable antibody‐loaded supramolecular nanofiber hydrogel has shown potential in inhibiting retinal vascular proliferation, attenuating choroidal neovascularization, and reducing ROS levels.^[^ ^79^ ^]^ Overall, hydrogels represent an innovative approach for ocular drug delivery, improving drug efficacy and patient compliance.

#### 3.2.3 Nanotubes

Carbon nanotubes (CNTs) are cylindrical structures composed of carbon atoms that have unique properties, such as electrical and thermal conductivity, strength, and a high surface area‐to‐volume ratio, making them valuable in the medical field.^[^ ^80^ ^]^ CNTs can be divided into single‐walled and multiwalled carbon nanotubes.^[^ ^81^ ^]^ Their surfaces are naturally hydrophobic, which can be addressed through functionalization, involving the introduction of desired functional groups onto the CNT walls to increase their solubility and biocompatibility. Functionalization can involve covalent bonding,^[^ ^82^ ^]^ the formation of strong chemical bonds, or noncovalent bonding,^[^ ^83^ ^]^ where therapeutic molecules are attached via adsorption. Each method has implications for the resulting drug delivery system. Due to their large surface area, functionalized CNTs exhibit a high drug‐loading capacity and have been used in applications such as tablet coating and targeted drug delivery for various disorders. They also have potential for use in ocular drug delivery, although this property has not been extensively explored.

#### 3.2.4 Dendrimers

Dendrimers are nanosized (usually 2–100 nm), symmetrical, and typically tree‐shaped hyperbranched structures that allow high capacities of drug encapsulation and conjugation.^[^ ^84^ ^]^ They can be functionally modified for various biomedical applications, including drug and nucleic acid delivery. For example, astodrimer sodium (SPL7013) is a polyanionic dendrimer with antiviral activity that effectively treats adenoviral eye infections, as indicated by Romanowski et al.^[^ ^85^ ^]^ Kambhampati et al. discovered that dendrimer–triamcinolone acetonide conjugates could be a novel approach for treating AMD, significantly inhibiting choroidal neovascularization.^[^ ^86^ ^]^ Additionally, Wang and colleagues developed dendrimer gel particles (DHPs) that combine the advantages of dendrimers, hydrogels, and nanoparticles.^[^ ^87^ ^]^ These DHPs demonstrated enhanced drug delivery efficiency, introducing a new possibility for combining multiple nanotechnologies in precision drug delivery.

#### 3.2.5 Liposomes

Liposomes are lipid vesicles composed of phospholipid bilayers that can encapsulate both hydrophilic and lipophilic drugs, playing a significant role in retinal disease treatment.^[^ ^88^ ^]^ The FDA‐approved verteporfin liposome, which is used to treat AMD, is an example of a liposome application.^[^ ^89^ ^]^ Liposomes can adhere to the cornea, facilitating the delivery of drugs with high molecular weights and poor absorption, aided by their positive charge binding to the cornea's negatively charged mucin coating.^[^ ^51^ ^]^ This feature also enhances corneal permeability, as observed for positively charged liposomes and penicillin G. Research on the size, surface charge, and surface coating of liposomes reveals that smaller liposomes can penetrate the retina, whereas larger liposomes cannot, highlighting the importance of the particle size. Additional factors, such as PEGylation and an anionic surface charge, also affect the retinal liposome distribution. Despite their potential, including the use of cationic liposome eye drops loaded with tacrolimus for dry eye treatment, limitations such as the drug loading capacity, short shelf‐life, and sterilization issues remain.^[^ ^45a^ ^]^

#### 3.2.6 Polymeric Micelles

Polymeric micelles (PMs) are self‐assembled nanostructures known for encapsulating and delivering hydrophobic drugs.^[^ ^90^ ^]^ The core‒shell structure consists of a hydrophobic core that carries the drug, while the outer shell, or corona, protects it from the aqueous environment. The properties of PMs, such as their high loading capacity, enhanced permeability, and potential for modifying their physicochemical characteristics, make them ideal for drug delivery.^[^ ^91^ ^]^ PM carriers can be synthesized using three methods: micelle‐forming polymer‒drug conjugates, polymeric micellar nanocontainers, and polyion complex micelles.^[^ ^92^ ^]^ Drug release from PMs involves two pathways: micelle dissociation and drug‒polymer bond breakage. PMs are composed of various polymeric segments, with some forming a hydrophilic outer shell and others forming a hydrophobic core. PMs have significant applications in oral chemotherapy because of their bioadhesive properties and ability to inhibit efflux transporters. Additionally, the targeted drug delivery capabilities of PMs are enhanced by increasing drug permeability through biological membranes.

#### 3.2.7 Metallic Nanostructures

Metallic nanostructures, particularly gold, silver, gadolinium, and iron oxide nanoparticles, hold significant potential in targeted drug delivery systems (DDSs).^[^ ^93^ ^]^ These systems can carry large drug doses, with drug loading achieved through noncovalent interactions. They can be functionalized through various methods for different biological applications. Gold nanoparticles, in particular, are valuable for drug and gene delivery and can be synthesized in various forms and sizes, even using microorganisms. Biocompatibility studies have shown that cells can take up gold nanoparticles without cytotoxic effects. Additionally, these metallic nanoparticles are beneficial for treatment due to their stability, half‐life, and ability to target specific sites. Researchers have developed targeted gold nanoparticles encapsulated with a thiol‐modified PEG coating for targeted action. Finally, a ferric–ferrous mixture of nanoparticles has shown promise in delivering erythropoietin to injured sites in the central nervous system, guided by magnetic control without neurotoxicity.^[^ ^94^ ^]^

#### 3.2.8 Quantum Dots

Quantum dots (QDs) are a type of nanoparticle with a crystalline structure composed of a semiconductor core, a shell, and a cap. They possess unique optical characteristics and are typically small, making them valuable in biomedical research, particularly for applications such as traceable drug delivery and theranostics (combined therapy and diagnostics). QDs can also be modified with tailored peptides or other molecules for targeted delivery to specific organs.^[^ ^95^ ^]^ They should have certain properties, such as nonreactivity with the drug, a high drug capacity, efficient encapsulation, good biocompatibility, low cytotoxicity, mechanical strength, and stability. Current research has focused on the use of quantum dots with less toxic cores, such as indium phosphate and zinc acetate.^[^ ^96^ ^]^ Graphene QDs have also attracted interest owing to their excellent biocompatibility and ability to be transported through biological membranes.^[^ ^97^ ^]^ Quantum dot synthesis techniques include top‐down processing methods and bottom‐up or self‐assembly techniques. The top‐down approach involves reducing a broad semiconductor into finer components via electron beam lithography, wet chemical methods, or reactive‐ion etching. In contrast, bottom‐up methods use precipitation tactics, including sol‐gel and microemulsion methods, among others. Certain strategies also utilize a vapor‐phase process in which layers are grown at the atomic level to produce QDs. This method is predominantly used to generate QDs derived from III‐V and II‐IV semiconductors.

### 3.3 Methods for Preparing High‐Dose Nanomaterials

Three primary techniques are used to develop nanoparticles with high drug‐loading capabilities: postloading, coloading, and preloading (Figure 8b). Due to the unique properties (such as the material type, structure, and surface characteristics) of nanoparticles, an appropriate strategy can be selected to achieve high drug loading.

In the postloading approach, the first step is to fabricate the nanocarriers. These nanocarriers often have porous structures, such as those found in silica nanoparticles,^[^ ^98^ ^]^ metal–organic framework (MOF) nanoparticles,^[^ ^99^ ^]^ iron nanoparticles,^[^ ^100^ ^]^ carbon nanoparticles,^[^ ^101^ ^]^ and hydrogel nanoparticles,^[^ ^102^ ^]^ and others, including magnesium silicate,^[^ ^103^ ^]^ calcium silicate hydrate,^[^ ^104^ ^]^ and hydroxyapatite.^[^ ^105^ ^]^ These porous materials inherently have a large surface area, adjustable pore size and volume, and straightforward chemistry for functionalization.^[^ ^106^ ^]^ Additionally, nonporous materials such as proteins and polypeptides have been investigated as candidates for the postloading strategy.^[^ ^107^ ^]^ Drug molecules can be loaded onto these nonporous carriers through noncovalent hydrophobic interactions, electrostatic attachment, hydrogen bonding, and *π*–*π* stacking. Nanoparticles developed using this method can achieve drug loading efficiencies ranging from 11.8% to 68.1%.

Coloading refers to a technique in which a drug is incorporated or encapsulated during nanoparticle construction. Numerous systems have been engineered using this strategy, including drug‒polymer composites,^[^ ^108^ ^]^ unaltered drugs,^[^ ^109^ ^]^ proteins,^[^ ^110^ ^]^ MOFs with embedded drugs,^[^ ^111^ ^]^ drug‒silsesquioxane mixtures,^[^ ^112^ ^]^ solid lipids,^[^ ^113^ ^]^ and polymers.^[^ ^114^ ^]^ For systems involving conjugation, covalent binding is relevant, whereas noncovalent interactions such as hydrophobic, electrostatic, and π‐π interactions play significant roles in polymers and proteins. The coloading strategy has successfully achieved drug loading values ranging from 18.5% to 100% and has produced particles with sizes between 29 and 400 nm.

In the preloading approach, drug nanoparticles are first generated, followed by the construction of a shell that protects and stabilizes the drug core. By adjusting the shell thickness, high‐drug‐loading nanoparticles can be created. The core–shell structure offers several advantages: it acts as a diffusion barrier controlling drug release, provides a shield for the drug core against external degradation, and the shell itself can be engineered to achieve variable release rates (i.e., sustained or stimuli‐responsive release).^[^ ^115^ ^]^ Additionally, the shell can be modified for various biological applications by attaching targeting components, linkers, spacers, or other functional entities useful for diagnostics, drug delivery, imaging, biosensing, etc. Polymers are the preferred shell material for most nanoparticle systems produced using this strategy, primarily due to their excellent biocompatibility, biodegradability, and ease of creation, although other materials, such as silica and lipids, have also been explored. Drug loadings achieved using the preloading technique range from 12.0% to 78.5%, resulting in nanoparticles varying in size from 40 to 984 nm.

### 3.4 Methods for Preparing Multifunctional Responsive Nanomaterials

Adjustable drug release is essential in nanotherapy because it provides greater control over the therapeutic index, thereby improving efficacy and minimizing side effects. This property allows the precise regulation of dosing, ensuring that the drug is released at the desired rate and concentration. This adjustable drug release can maintain a consistent therapeutic level in the body, providing effective treatment over time and avoiding the peaks and troughs often associated with conventional dosing schedules. Adjustable drug release also enhances patient compliance by potentially reducing the frequency of drug administration, thereby significantly improving the quality of life for patients on long‐term medication. Therefore, tunable drug release is a critical component in the effective and efficient application of nanotherapies.

The effectiveness of payload delivery relies on the efficiency of drug packaging and the design of release mechanisms within the nanosystems. The efficiency of “packaging” drugs is determined by the encapsulation or drug conjugation techniques used. Different nanoparticles offer various methods for entrapping drug molecules, which will be discussed further. Modulating the speed of drug release in response to a trigger is a key strategy for controlling drug release and maintaining an effective therapeutic dose over time. Nanosystems can be classified into two groups based on the triggers that stimulate drug release, open‐loop control systems and closed‐loop control systems, as outlined in Figure 8c. In open‐loop systems, external factors such as magnetic pulses, heat, sound pulses, or electrical fields regulate drug release. Conversely, in closed‐loop systems, the drug release rate is synchronized with the presence and intensity of internal stimuli at the target areas.^[^ ^116^ ^]^ Current strategies generally focus on the internal “chemistry” of nanosystems that respond to pH or temperature, erosion due to the local chemical environment, redox reaction‐based release, and enzyme‐mediated release, as discussed below.^[^ ^117^ ^]^

In the redox‐activated drug release process, a redox‐reactive nanocarrier with functional groups is used. These functional groups respond when they contact the reducing or oxidizing environment present in and around cancer cells (rich in agents such as GSH, free radicals, and peroxides), leading to the breaking of chemical bonds. This chemical transformation can, in turn, trigger changes in the hydrophobic properties of the polymer, destabilizing the nanoparticles and triggering the release of the drug payload. For example, poly(propylene sulfide) polymer nanoparticles use disulfide bonds as redox‐sensitive elements. When these bonds react with H_2_O_2_, a shift in the hydrophobicity of the polymers is triggered, causing the nanoparticles to disintegrate and the drug to be released.^[^ ^118^ ^]^ Remarkably, redox‐responsive units may also exhibit a nonlinear reaction to stimuli. This complexity allows for a swift response to high concentrations of stimuli but not to low concentrations, thereby achieving a certain level of controlled specificity.^[^ ^119^ ^]^

The acidic microenvironment outside of cancerous cells is primarily due to the release of lactic acid from glycolysis, which causes the pH to decrease to ≈6.5—a significant decrease from the typical physiological pH of 7.4—during tumor metastasis or growth.^[^ ^120^ ^]^ This pH difference between healthy and pathological cells provides an opportunity for regulating drug distribution. Various chemical bonds have been explored to facilitate the drug dispensation process. Covalent bonds sensitive to pH changes, including benzoic‐imine bonds, the 1,3,5‐triazaadamantane group, and hydrazone bonds, have been developed as initial systems with pH‐responsive mechanisms.^[^ ^121^ ^]^ Gao and colleagues used benzoic‐imine bonds to attach α‐cyclodextrin to mesoporous silica nanoparticles, which undergo partial hydrolysis in the extracellular space of tumors and full hydrolysis within endosomes at a low pH of ≈5. Researchers then assessed the step‐by‐step release of the loaded drug doxorubicin in environments with various pHs to validate the system's efficacy.^[^ ^122^ ^]^

Controlled drug release has been explored using various stimuli, such as heat production under a magnetic field,^[^ ^123^ ^]^ electrochemically triggered drug distribution systems,^[^ ^124^ ^]^ photoactivatable systems,^[^ ^125^ ^]^ and ultrasound‐triggered systems.^[^ ^126^ ^]^ Given the ongoing progress in molecular biology and enzyme engineering, the application of chemical methods for particle surface modification or functionalization for specificity is limitless. Methods for integrating and conjugating biomolecules will contribute to the creation of well‐regulated drug delivery systems, addressing the deficiencies observed in existing systems.

## 4 Nanotherapy‐Based Vision Restoration Strategies

### 4.1 Drug Delivery

Nanotechnologies are revolutionizing drug delivery methods by increasing bioactivity and improving drug bioavailability at targeted sites, particularly in the retina.^[^ ^127^ ^]^ Nanoparticle‒drug combinations offer continuous drug release, prolonged retention, and targeted delivery to specific retinal sites.^[^ ^128^ ^]^ Various strategies have been proposed to address challenges in the precise placement of administered cells, including the use of hydrogels or carrier systems and techniques to prevent cell death due to reflux.^[^ ^129^ ^]^

**Figure 9:**
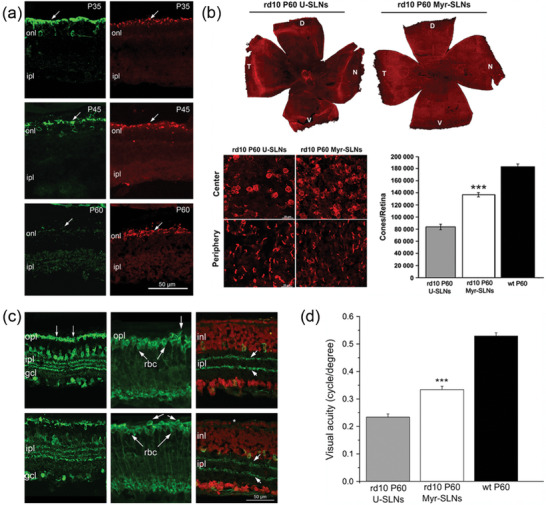
a) Survival of rods (green) and cones (red) in retinas of rd10 mice treated with myriocin. Rhodopsin staining (green signal) at 35, 45, and 60 days of age. The staining in the outer segments is still clearly visible at P35, becomes irregular and clustered at P45 and is virtually absent by P60. Cone‐opsin staining (red signal) is evident at both P35 and P60. A continuous row of opsin‐positive cells is still present at P60. These are cones in which outer segments are very short; however, the staining is concentrated in cell bodies. onl, outer nuclear layer; ipl, inner plexiform layer. Scale bar: 50 µm. Reproduced with permission.^[^ ^133^ ^]^ Copyright 2013, Federation of European Neuroscience Societies and Blackwell Publishing Ltd. b) Comparison of the survival of cones in unloaded solid lipid nanoparticles and myriocin‐loaded solid lipid nanoparticles at P60. Low magnification of whole‐mount retinas stained with antibodies against cone‐specific opsins (red; blue and red/green cone‐opsins). An area devoid of cones is evident in the central part of the retina treated with U‐SLNs, whereas cones are still preserved in Myr‐SLN‐treated retina. D, V, N, T, dorsal, ventral, nasal and temporal poles of the retina. High‐magnification confocal images of retinas stained for cone‐opsins obtained from central and peripheral regions of the samples in A. Counts of cones in retinas from U‐SLN‐treated, Myr‐SLN‐treated (n = 5 per condition) and wt mice (n = 3). Student's t‐test on rd10 mice, ***p = 0.0001. Reproduced with permission.^[^ ^133^ ^]^ Copyright 2013, Federation of European Neuroscience Societies and Blackwell Publishing Ltd. c) Inner retinal neurons in myriocin‐treated and control‐treated retinas. (A and D) Calbindin staining of horizontal, amacrine and ganglion cells. (B and E) Protein kinase C staining of rod bipolar cells (rbc). The dendrites of these neurons are more clearly visible in the myriocin‐treated preparation (arrow in B) than in the control specimen. Also, cell bodies of rod bipolar cells are more frequently displaced in the outer retina of the sample in E compared with B (double arrows in E). (C and F) Cholinergic amacrine cells (green signal) and nuclear counterstaining (red signal). The two cholinergic bands can be appreciated clearly in both specimens (pair of arrows in C and F). In F (untreated retina), the discontinuous aspect of the photoreceptor layer is evident as many photoreceptors are missing (asterisk in F). opl, outer plexiform layer; ipl, inner plexiform layer; gcl, ganglion cell layer; inl, inner nuclear layer. Scale bar: 50 µm. Reproduced with permission.^[^ ^133^ ^]^ Copyright 2013, Federation of European Neuroscience Societies and Blackwell Publishing Ltd. d) VA of U‐SNL‐treated (*n* = 10), Myr‐SNL‐treated (*n* = 9) rd10 and wt mice (*n* = 4) at P60. Student's *t*‐test on rd10 mice, ***p = 0.0001. Reproduced with permission.^[^ ^133^ ^]^ Copyright 2013, Federation of European Neuroscience Societies and Blackwell Publishing Ltd.

One of the primary targets of these treatments is retinal pigment epithelial (RPE) cells, which are located between the choroid and the neural retina.^[^ ^130^ ^]^ Conditions such as dry age‐related macular degeneration (AMD) can damage RPE cells, impairing their ability to support photoreceptor cells and eventually leading to vision loss.^[^ ^131^ ^]^ Nanobased cell transplantation has emerged as an innovative alternative to nanotherapeutics for treating neovascular conditions, representing a promising approach for transplanting donor or allogeneic RPE cells.^[^ ^132^ ^]^ Additionally, Piano et al.^[^ ^133^ ^]^ reported that the noninvasive topical (transcorneal) application of solid lipid nanoparticles loaded with myriocin increased photoreceptor survival by reducing retinal ceramide levels in an rd10 mouse model (**Figure** **9**).^[^ ^134^ ^]^

**Figure 10:**
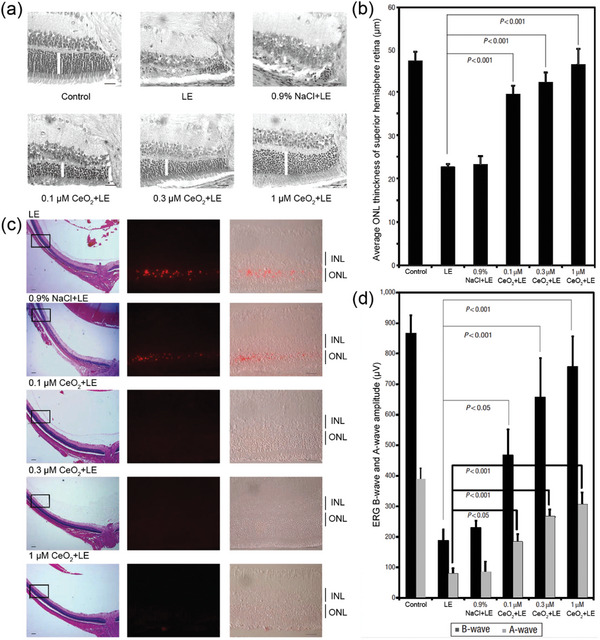
a) Intravitreal injection of nanoceria particles protects rat retina photoreceptor cells from light‐induced degeneration. Intravitreal injection of nanoceria particles protects rat retina photoreceptor cells from light‐induced degeneration. Nanoceria particles provide pan‐retinal protection against light damage. Reproduced with permission.^[^ ^137^ ^]^ Copyright 2006, Springer Nature Limited. b) Nanoceria particles prevent the appearance of TUNEL‐positive photoreceptor cells, which occurs days after exposure to damaging light. Retinal function is protected by the nanoceria particles in a dose‐dependent manner. Copyright Nature Publishing Group. Reproduced with permission.^[^ ^137^ ^]^ Copyright 2006, Springer Nature Limited.

Among various nanometals, cerium oxide nanoparticles (nanoceria) stand out as among the most promising materials in biomedical engineering for treating retinal diseases.^[^ ^135^ ^]^ Due to their redox‐active radical‐scavenging properties (antioxidant activity),^[^ ^136^ ^]^ several nanoceria‐based therapeutic formulations have been developed for ophthalmic applications, particularly for retinal regeneration. The oxidation state of nanoceria changes when oxygen or its electrons are lost, creating defects in the lattice structure. As the size of nanoceria decreases (3–5 nm diameter), more oxygen vacancies are present in the crystal structure, increasing its antioxidant capabilities.^[^ ^18b^ ^]^ Chen et al. showed that nanoceria could prevent retinal damage associated with oxidative stress in an animal model of retinal angiomatous proliferation, and an intravitreal injection of nanoceria has been shown to prevent light‐induced photoreceptor damage in rodents, even when it is administered after the initiation of light damage (**Figure** **10**).^[^ ^137^ ^]^ Thus, nanoceria may be effective in treating the complications of AMD and diabetic retinopathy (DR). Furthermore, previous in vivo studies have shown that nanoceria can prevent long‐term retinal neurodegeneration without side effects.^[^ ^138^ ^]^ In addition to preventing damage, several experimental studies have revealed the effectiveness of nanoceria in different formulations for treating retinal damage, such as acute damage due to exposure to high‐intensity light.^[^ ^139^ ^]^

The long‐term safety of nanoceria in ophthalmic tissue has been documented in rats, with no adverse effects on the function or cytoarchitecture of their retinas.^[^ ^140^ ^]^ Due to its nanoscale size, nanoceria can be administered topically through corneal permeation via PEGylation and liposomal encapsulation strategies without altering its physicochemical properties, making it a strong candidate for treating various posterior segment eye disorders.^[^ ^141^ ^]^ However, the most common method of drug delivery for treating vitreoretinal diseases is intravitreal injection into the posterior segment.^[^ ^142^ ^]^ This method was successfully employed by Wong et al. to deliver Alexa Fluor 647‐conjugated nanoceria to the vitreous of both male and female BALB/c mice.^[^ ^143^ ^]^ A delay in disease progression was observed after a single intravitreal injection of inorganic antioxidant catalytic nanoceria, confirming its suitability for retinal treatment. Moreover, although the Alexa Fluor 647‐conjugated nanoceria remained in the retina for more than a year, the specific retinal cell types that preferentially take up nanoceria have yet to be identified. Zhou et al. developed vacancy‐engineered nanoceria that can inhibit the development and promote the regression of pathological retinal neovascularization in very low‐density lipoprotein receptor (Vldlr) knockout mice, which carry a loss‐of‐function mutation in the Vldlr gene and exhibit phenotypes similar to retinal angiomatous proliferation.^[^ ^138^ ^]^ Regression occurs even if intravitreal nanoceria treatment is administered after the mutant retinal phenotypes are established. A single injection has a prolonged effect (weeks) because nanoceria is both a catalytic and regenerative antioxidant. Additionally, nanoceria inhibits the development of increased vascular endothelial growth factor (VEGF) levels in this model, suggesting potential effectiveness in treating diabetic macular edema and choroidal neovascularization‐induced retinal edema in AMD eyes.

**Figure 11:**
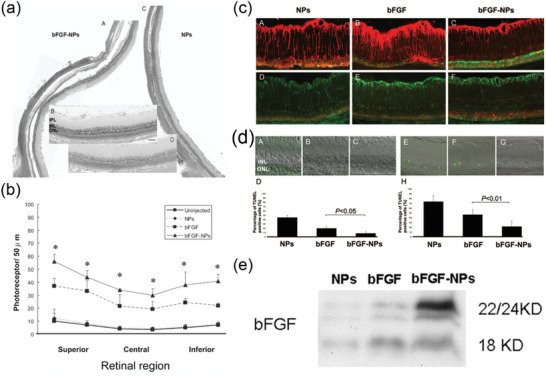
a) Morphologic rescue of the superior retina of RCS rats. Reproduced with permission. Copyright 2007, ARVO Journals. b) Mean number of photoreceptors per 50 um for all treatment groups of RCS rats 8 weeks after injection. Reproduced with permission.^[^ ^144^ ^]^ Copyright 2007, ARVO Journals. c) Immunohistochemistry of the retina of RCS rats 8 weeks after injection. Reproduced with permission.^[^ ^144^ ^]^ Copyright 2007, ARVO Journals. d) Fluorescence photomicrographs of retinal sections from TUNEL assay at 4 (A‐C) and 8 weeks (E‐G) after injection. (A, E) BlankNP‐treated retina; (B, F) bFGF‐treated retina; (C, G) bFGF‐NP‐treated retina. The percentage of TUNEL‐positive cells in the ONL is smaller in the bFGF‐NP‐treated retina than in the bFGF‐treated retina 4 and 8 weeks after injection (D, H). Scale bar, 50 µm. Reproduced with permission.^[^ ^144^ ^]^ Copyright 2007, ARVO Journals. e) Expression of bFGF protein in 9‐week‐old RCS rats by Western blot analysis; bFGF expression level was greater in the bFGFNP‐treated retina than in the bFGF‐treated retina. Reproduced with permission.^[^ ^144^ ^]^ Copyright 2007, ARVO Journals.

Sakai et al. prepared basic fibroblast growth factor‐impregnated nanoparticles (bFGF‐NPs) to treat retinal degenerative disease in Royal College of Surgeons (RCS) rats.^[^ ^144^ ^]^ These rats have a mutation in a tyrosine kinase that prevents proper outer segment phagocytosis by RPE cells, leading to progressive rod and cone photoreceptor degeneration. Some forms of retinitis pigmentosa (RP) in humans arise from this same mutation.^[^ ^145^ ^]^ Sakai et al.^[^ ^144^ ^]^ reported consistently greater opsin preservation in bFGF‐NP‐treated retinas, a significantly greater number of photoreceptors, significantly fewer TUNEL‐positive cells, and a significant increase in bFGF levels in treated retinas, verifying that bFGF‐NPs can provide sustained retinal rescue (**Figure** **11**).

### 4.2 Gene Delivery

Gene replacement therapy using retinal‐tropic nanoparticles represents a significant advance in gene transfer for both preclinical and clinical research.^[^ ^146^ ^]^ Generally, delivery systems can be classified into two types: viral gene delivery and nonviral gene delivery. In clinical applications, multiple viral nanoparticles are used to transduce target cells with excellent efficiency.^[^ ^147^ ^]^ However, other nanoparticles, such as lenti‐ and adenovirus‐based nanoparticles,^[^ ^148^ ^]^ have also been investigated for gene delivery to the retina but have shown insufficient efficiency in photoreceptor gene transduction.^[^ ^149^ ^]^ To date, most of these studies have been conducted in mouse models, which have greatly aided in the development and optimization of treatment strategies.

Gene delivery systems for eye diseases range from simple eye drops and ointments to more advanced nanotechnology‐based systems, such as nanoparticles, dendrimers, mucoadhesive systems, iontophoresis, ocular inserts, and viruses.^[^ ^150^ ^]^ Nonviral gene delivery employs diverse cationic lipids and polymers that compact and bind therapeutic DNA into nanosized particles.^[^ ^151^ ^]^ Nonviral gene‐delivery nanoparticles have several advantages over viral nanoparticles, including easier and less expensive manufacturing, reduced immunogenicity, and flexibility in the size of the transgene delivered.^[^ ^152^ ^]^ However, nonviral alternatives have been plagued by low transfection efficiency, short‐term expression, and low expression levels.^[^ ^153^ ^]^ These approaches are currently less efficient than the use of viral nanoparticles and require significant development before they can be used clinically.^[^ ^154^ ^]^

Recently, these drawbacks have been overcome using specialty carriers such as polylysine,^[^ ^155^ ^]^ liposomes, or polyethyleneimines and the inclusion of suitable DNA elements to increase gene expression and longevity. Efforts have yielded nonviral nanoparticles with favorable safety profiles, a lack of immunogenicity, long‐term elevated gene expression, and efficient transfection in the retina and RPE, indicating their potential for clinical applications. Researchers have reported that nonviral nanoparticles can be used to deliver RS1‐ or RPE65‐expressing plasmids into the retina.^[^ ^156^ ^]^

**Figure 12:**
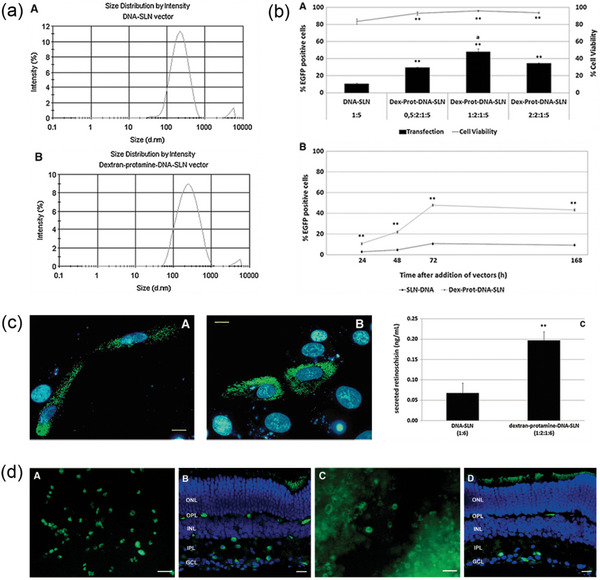
a) Size distribution of the DNA‐SLN vector and dextran‐protamine‐DNA‐SLN vector. Reproduced with permission.^[^ ^156a^ ^]^ Copyright 2012, Mary Ann Liebert, Inc. b) Transfection (columns) and cell viability (line) for each formulation assayed in ARPE‐19 cells at 72 h, in which the SLN‐to‐DNA ratio (w/w) was 5:1 in all cases and the dextran‐protamine‐DNA ratio varied from 0.5:2:1 to 2:2:1; Transfection of ARPE‐19 cells by nonviral vectors overtime. The SLN‐to‐DNA ratio (w/w) was 5:1 and the dextran‐protamine‐DNA‐SLN ratio was 1:2:1:5. Error bars represent the SD (*n* = 3). **p < 0.01 with respect to the DNA‐SLN formulation. ^a^p<0.01 with respect to all other formulations. Reproduced with permission.^[^ ^156a^ ^]^ Copyright 2012, Mary Ann Liebert, Inc. c) Detection of retinoschisin expressed in ARPE‐19 cells by fluorescence microscopy after transfection with dextran‐protamine‐DNA‐SLN vector (1:2:1:6) and DNA‐SLN vector (1:6), and Quantification of secreted retinoschisin shows the difference. Delgado D, del Pozo‐Rodríguez A, Solinís MÁ, Avilés‐Triqueros M, Weber BH, Fernández E, Gascón AR. Reprinted from Dextran and protamine‐based solid lipid nanoparticles as potential vectors for the treatment of X‐linked juvenile retinoschisis Reproduced with permission.^[^ ^156a^ ^]^ Copyright 2012, Mary Ann Liebert, Inc. d) pCMS‐EGFP expression in the retina 72 h after transfection with dextran‐protamine‐DNA‐SLN vector. (A and C) Whole‐mount retinas. (B and D) Cryostat sections with cell nuclei counterstained with Hoechst (blue). Note the prominent localization of pCMS‐EGFP in the inner retinal layers after a single intravitreal injection (A and B), whereas with subretinal injection the transfection can be seen in several layers, primarily in the retinal pigment epithelium and photoreceptors but also in processes at the outer plexiform layer and in some retinal ganglion cells (C and D). ONL, outer nuclear layer; OPL, outer plexiform layer; INL, inner nuclear layer; IPL, inner plexiform layer; GCL, ganglion cell layer. Scale bars: 25 lm. Color images available online at www.liebertonline.com/hum. Reproduced with permission.^[^ ^156a^ ^]^ Copyright 2012, Mary Ann Liebert, Inc.

Delgado et al. analyzed the potential application of nonviral nanoparticles based on solid lipid nanoparticles (SLNs), dextran, protamine, and a plasmid (pCMS‐EGFP or pCEP4‐RS1) for gene therapy for X‐linked juvenile retinoschisis.^[^ ^156a^ ^]^ They reported that the presence of dextran and protamine in SLNs greatly increased the expression of retinoschisin and increased the level of green fluorescent protein (EGFP) in ARPE‐19 cells. Transfection was observed in several retinal layers after ocular administration in Wistar rats, demonstrating the efficacy of SLNs (**Figure** **12**). In a similar experiment using compacted DNA nanoparticles containing a plasmid with a scaffold/matrix attachment region (S/MAR) and the vitelliform macular dystrophy 2 promoter (VMD2‐P), Koirala et al. tested the ability of these nanoparticles to target the RPE, drive long‐term, tissue‐specific gene expression, and mediate proof‐of‐principle rescue in the rpe652/2 model of Leber congenital amaurosis (LCA). They reported that the S/MAR‐containing plasmid presented highly persistent reporter gene expression at relatively high copy numbers.^[^ ^157^ ^]^

**Figure 13:**
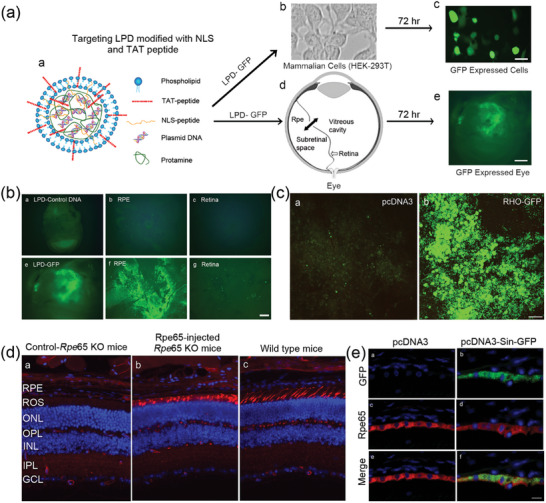
a) LPD‐mediated gene delivery into mammalian and retinal cells. Schematic illustration of targeting LPD modified with NLS and TATpeptide complexed with GFP to HEK‐293T mammalian cells or by subretinal injection into eye. Reproduced with permission.^[^ ^158^ ^]^ Copyright 2014, American Chemical Society. b) Plasmid DNAs of either control (pcDNA3 vector) or GFP were complexed with LPD and injected subretinally into BALB/c mice. Eyes were removed and examined for GFP expression under inverted fluorescence microscopy; and whole RPE, retinal flat mounts were prepared and examined for GFP expression under inverted fluorescence microscopy. Reproduced with permission.^[^ ^158^ ^]^ Copyright 2014, American Chemical Society. c) LPD‐mediated retinal specific expression by mouse rhodopsin promoter. Plasmid DNAs of either control (pcDNA3 vector) or Rhodopsin‐GFP (RHO‐GFP) were complexed with LPD and injected subretinally into BALB/c mice. One week later, retinal flat mounts were prepared and examined for GFP expression under confocal microscopy. Scale bar: 50 µm. Reproduced with permission.^[^ ^158^ ^]^ Copyright 2014, American Chemical Society. d) LPD‐mediated Rpe65 gene delivery to *Rpe65* knockout mice rescues cone cell death. Retinal sections prepared from uninjected and Rpe65‐injected *Rpe65* knockout and wild type mice were subjected to lectin cytochemical analysis using peanut agglutinin. RPE, retinal pigment epithelium; ROS, rod outer segments; ONL, outer nuclear layer, OPL, outer plexiform layer, INL, inner nuclear layer, IPL, inner plexiform layer, GCL, ganglion cell layer. Scale bar: 100 µm. e) In vivo Rpe‐specific delivery of GFP by LPD. Plasmid DNAs of either control (pcDNA3 vector) or Sin‐GFP were complexed with LPD and injected subretinally into BALB/c mice. One week later, retinal sections were prepared and examined for GFP expression under inverted fluorescence microscopy or by costaining the sections with Rpe65 antibody. Panels e and f represent the merged images of GFP and Rpe65. Nuclei were stained with DAPI (blue). Scale bar: 20 µm. Reproduced with permission.^[^ ^158^ ^]^ Copyright 2014, American Chemical Society.

This plasmid was selected for testing in the rpe652/2 mouse model to determine whether nanoparticles or the VMD2‐hRPE65‐S/MAR plasmid could lead to structural and functional improvements in the LCA disease phenotype. The results revealed that nonviral delivery of DNA nanoparticles (hRPE65) can result in persistent, therapeutically efficacious gene expression in the RPE. Rajala et al. developed an artificial viral nanoparticle using a liposome‒protamine‒DNA complex (LPD) modified with a cell‐permeable peptide and a nuclear localization signal (NLS) peptide.^[^ ^158^ ^]^ These nonviral nanoparticles carrying the RPE65 gene were administered subretinally in mice lacking RPE65, resulting in long‐term gene expression and better preservation of cones (**Figure** **13**). Similarly, Thomson et al. presented a method for modifying cationic gene nanomedicines with hyaluronic acid to improve retinal drug delivery efficiency after intravitreal administration.^[^ ^159^ ^]^ They prepared poly(lactic‒coglycolic acid) (PLGA) scaffolds with different copolymer ratios for regenerating the RPE and reported that increasing the polylactic acid ratio in the PLGA polymer resulted in a less porous structure and denser scaffolds.^[^ ^160^ ^]^ Low‐molecular‐weight hyaluronic acid (LMWHA)‐coated nanopolyplexes are taken up in vitro by retinal target cells, presumably via CD44‐mediated endocytosis. As an electrostatic coating for cationic binary PECs, LMWHA is considered a biocompatible material that enhances the efficiency of gene nanomedicine delivery to the retina via intravitreal administration. Batabyal et al.^[^ ^161^ ^]^ introduced a continuous wave near‐infrared laser‐based nano‐enhanced optical delivery method for the spatially controlled delivery of opsin‐encoding genes into the retina in vivo. Optical field enhancement by gold nanorods is utilized to transiently permeabilize the cell membrane, enabling the delivery of exogenous impermeable molecules to nanorod‐binding cells in laser‐irradiated regions.

Finally, polyethylene glycol‐substituted lysine peptide (CK30PEG)‐compacted DNA nanoparticles have been used in vivo to drive gene expression and mediate therapeutic rescue in the retina^[^ ^162^ ^]^ and RPE.^[^ ^162^, ^163^ ^]^ Retinal expression was several‐fold higher than that achieved with naked DNA^[^ ^42^, ^162^, ^164^ ^]^ and on the same scale as that observed with adeno‐associated viruses.^[^ ^165^ ^]^ In adult mice, a single subretinal injection of CK30PEG nanoparticles resulted in reporter gene expression in 55% of RPE cells within two days posttreatment.^[^ ^163^ ^]^ The subretinal delivery of these particles is safe and nontoxic.^[^ ^164^, ^166^ ^]^ Thus, CK30PEG nanoparticles are expected to be a viable tool for RPE‐based gene therapy.

Overall, ocular protection or restoration relies on treatments that promote neuronal regeneration.^[^ ^167^ ^]^ Gene therapy holds promising potential for providing neuroprotective and regenerative functions by delivering protective or antiapoptotic genes to injured cells. An ideal gene therapy method should have the following attributes: First, gene expression levels should be high enough to promote a phenotypic improvement without causing overexpression‐related toxicity. Second, gene expression should be sustained for long periods without the need for repeated administration. Third, the therapeutic load should not induce immunogenic or inflammatory responses in the host.^[^ ^168^ ^]^ Nevertheless, gene delivery nanoparticles generally lack efficiency in targeting the inner retina in humans due to their thick inner limiting membrane.^[^ ^169^ ^]^

### 4.3 Biocompatible Nanofunctional Scaffolds

Nanotechnology has enhanced the development of a new generation of prosthetic microdevices that provide biocompatible support, particularly subretinal implants. Nanoscaffolds have sufficient porosity for the infiltration of new cells while maintaining adequate mechanical strength,^[^ ^170^ ^]^ indicating a significant improvement over traditional membrane scaffolds. One of the most promising and widely studied techniques for producing nanoscaffolds is electrospinning. This technique allows the creation of nanoscaffolds from a variety of materials, including polymers and biomaterials. Researchers can fine‐tune specific characteristics of the resulting scaffold, such as the fiber diameter and surface morphology, according to the tissue requirements.^[^ ^171^ ^]^ Additionally, nanoscaffolds can closely mimic the size of extracellular matrix (ECM) proteins, which range from 50 to 500 nm in diameter.^[^ ^172^ ^]^ A consensus has been reached that nanoscaffolds perform better in terms of cell growth and transplantability than their membrane counterparts do.^[^ ^173^ ^]^ Enhancing the bioactivity of a scaffold can be achieved by modifying biomaterials to more closely mimic the ECM. This modification may involve integrating ECM‐derived bioactive molecules, such as signaling molecules, or incorporating features such as proteolytic degradation and cell adhesion enhancements before the scaffold is used in tissue engineering systems.^[^ ^174^ ^]^

**Figure 14:**
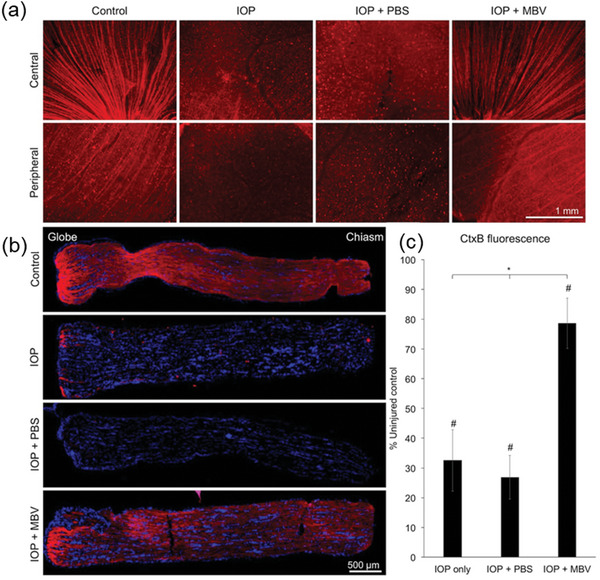
MBV decrease IOP‐induced axon degeneration. a) Representative images showing RGC axons labeled with cholera toxin subunit B (CTB, red) in the uninjured central and peripheral retina (Control), in IOP‐injured retina (IOP), and IOP‐injured retinas treated with either PBS (IOP+PBS) or MBV (IOP+MBV). Reproduced with permission.^[^ ^175^ ^]^ Copyright 2019, Nature. b) Representative images showing RGC axons labeled with CTB in the optic nerve of uninjured control eyes (Control), untreated IOP‐injured eyes (IOP), and IOP‐injured eyes treated with either PBS (IOP+PBS) or MBV (IOP+MBV). Reproduced with permission.^[^ ^175^ ^]^ Copyright 2019, Nature. c) Quantitatively, CTB immunofuorescence decreased in both untreated and PBS treated IOP‐injured optic nerves. However, in MBV treated IOP‐injured ONs, CTB was more similar to uninjured control animals. One‐way ANOVA with Post‐hoc Tukey's test determined significance between groups, *p < 0.05, and compared to uninjured control, #*p* < 0.05. Copyright 2019 scientific reports. Reproduced with permission.^[^ ^175^ ^]^ Copyright 2019, Nature.

Matrix‐bound nanovesicles (MBVs), a distinct class of extracellular nanovesicles localized specifically to the ECM of healthy tissues (**Figure** **14**),^[^ ^175^ ^]^ represent another promising approach. Van der Merwe et al. reported that MBVs can protect retinal ganglion cells (RGCs) and preserve visual function after severe intraocular pressure (IOP)‐induced ischemia in rats.^[^ ^175^ ^]^ Intravitreal injections of MBVs reduced IOP‐induced RGC axon degeneration and death, preserved RGC axon connectivity to visual nuclei in the brain, and prevented the loss of retinal function. In the optic nerve, MBVs also mitigated IOP‐induced decreases in growth‐associated protein‐43 expression and increases in glial fibrillary acidic protein expression. In vitro studies have shown that MBVs suppress proinflammatory signaling by activating microglia and astrocytes, stimulating RGC neurite growth, and protecting RGCs from neurotoxic stimuli produced by proinflammatory astrocytes. These findings suggest that MBVs can positively modulate distinct signaling pathways in diverse cell types. Since MBVs are naturally derived bioactive factors that are already present in numerous FDA‐approved devices, they hold potential as immunomodulatory and neuroprotective agents for treating retinal and other central nervous system (CNS) trauma or diseases.^[^ ^176^ ^]^

Similarly, biodegradable polycaprolactone (PCL) thin film scaffolds can be fabricated with integrated microtopography.^[^ ^177^ ^]^ Through coaxial electrospinning, researchers have developed core‒shell fibrous scaffolds from polyethylene glycol/polycaprolactone (PEG/PCL) with pigment epithelium‐derived factor (PEDF) encapsulated in the core. This flexible technique shows promise for promoting the photoreceptor differentiation of conjunctival mesenchymal stem cells seeded on these scaffolds.^[^ ^178^ ^]^ The core–shell scaffold loaded with PEDF (PEG+PEDF/PCL) offers superior control over the release profile of the factor, guiding mesenchymal stem cells to differentiate into photoreceptor cells and promoting regeneration.

**Figure 15:**
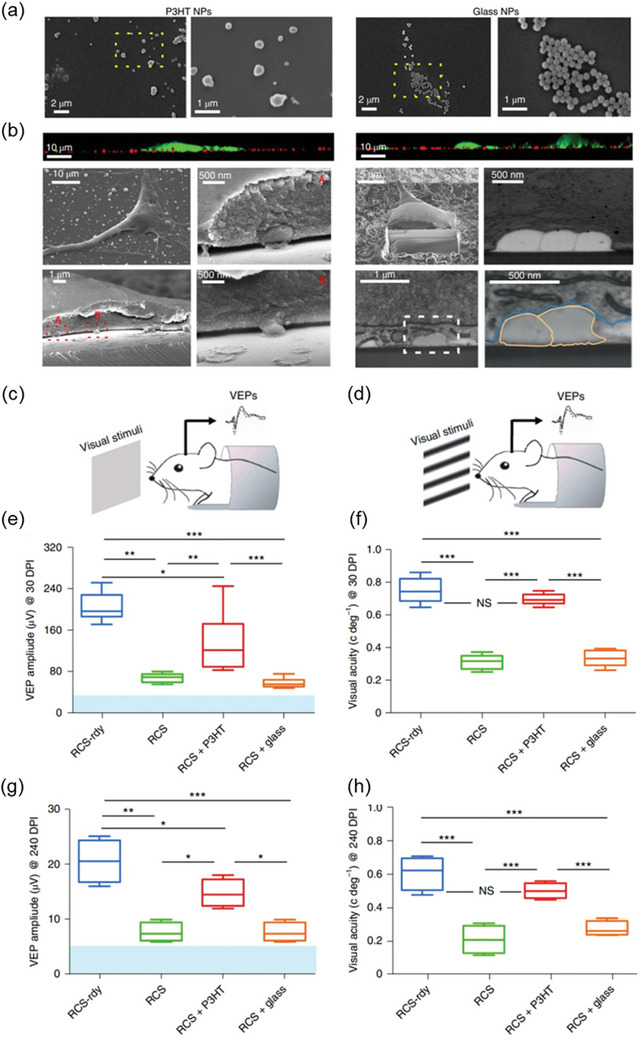
P3HT NPs form a tight seal with the neuronal membrane and trigger light‐evoked neuronal stimulation through a capacitive mechanism. a) Scanning electron microscope images of P3HT NPs generated with the reprecipitation method and control glass NPs of similar size. Reproduced with permission.^[^ ^180^ ^]^ Copyright 2020, Springer Nature Limited. b) Top: lateral projection of confocal z‐stack fluorescence images of calcein‐labelled primary neurons layered on coverslips drop‐cast with P3HT NPs. NPs (intrinsic red fluorescence) are visibly engulfed by the neuronal cell bodies and processes; bottom: ultrastructural analysis of neuron–NP interactions before and after sectioning of standard SEM preparations (left) and of resin‐embedded focused ion beam (FIB) cross sections (right). Reproduced with permission.^[^ ^180^ ^]^ Copyright 2020, Springer Nature Limited. c,d) Experimental set‐up for visual‐evoked‐potential (VEP) recordings in primary visual cortex in response to either flash stimuli (a; 20 cd m^−2^; 200 ms, 1 Hz) or horizontal sinusoidal gratings of increasing spatial frequency (b; 0.1 to 1 cycle per degree of visual angle) administered at 1 Hz. Reproduced with permission.^[^ ^180^ ^]^ Copyright 2020, Springer Nature Limited. e,g) VEP recordings in primary visual cortex in response to flash stimuli show a significant improvement of light sensitivity in RCS rats injected with P3HT NPs at both 30 and 240 DPI. Reproduced with permission.^[^ ^180^ ^]^ Copyright 2020, Springer Nature Limited. f,h) The electrophysiological analysis of VEP recordings in response to horizontal sinusoidal gratings reveals a full recovery of visual acuity (c deg^−1^, cycles per degree of visual angle) at both 30 and 240 DPI. NS, *P <  0.05, **P <  0.01, ***P <  0.001, one‐way ANOVA/ Tukey tests. Reproduced with permission.^[^ ^180^ ^]^ Copyright 2020, Springer Nature Limited.

An organic photovoltaic blend, specifically polymer (poly(3‐hexylthiophene) (P3HT)) nanoparticles, has been used for neuronal stimulation via a photoexcitation process.^[^ ^179^ ^]^ A recent study by Maya‐Vetencourt et al. showed that a subretinal injection of conjugated P3HT nanoparticles mediated light‐induced stimulation of retinal neurons without triggering trophic or proinflammatory effects (**Figure** **15**).^[^ ^180^ ^]^ Moreover, as a liquid retinal prosthesis, P3HT nanoparticles can rescue vision in a rat model of retinitis pigmentosa (RP).

### 4.4 Nano‐Mediated Stem Cell Regeneration

The incorporation of cells into scaffolds is a useful strategy for engineered tissue formation. Researchers are exploring the inclusion of stem cells in scaffolds to overcome the limitations of primary cells, such as their potential dysfunction.^[^ ^181^ ^]^ While stem cells hold promise for the development of new tissues, when transplanted alone, they often exhibit poor viability and low regenerative capabilities.^[^ ^182^ ^]^ These properties have driven further research into the development of tissue engineering scaffolds and systems.

One promising therapeutic strategy involves the replacement of retinal tissue lost to disease or trauma with retinal progenitor cells (RPCs) delivered on polymer scaffolds and transplanted into the subretinal space of the damaged retina.^[^ ^183^ ^]^ Several studies have documented improvements in stem and progenitor cell survival when these cells are delivered to the subretinal space on polymer scaffolds.^[^ ^184^ ^]^

Yao et al. developed biodegradable thin‐film PCL scaffolds with varying surface topographies using microfabrication techniques.^[^ ^185^ ^]^ Mouse retinal progenitor cells (mRPCs) cultured on PCL scaffolds exhibited an increased potential to differentiate toward a photoreceptor fate, suggesting that PCL scaffolds are promising substrates for guiding mRPC differentiation into photoreceptors in vitro before transplantation. When mRPC/PCL constructs were cocultured with retinal explants from rhodopsin‐null mice, mRPC/PCL constructs were associated with increased mRPC integration rates. Newly integrated mRPCs may localize to the outer nuclear layer, express appropriate markers of the photoreceptor fate, proliferate and express mature retinal proteins in response to interactions with nanowire scaffolds.^[^ ^186^ ^]^ These findings indicate that guided differentiation and organized delivery of mRPCs constitute a practical strategy for repairing damaged retinas.

Human induced pluripotent stem cells (hiPSCs) can be genetically reprogrammed into an embryonic stem cell‐like state for various medical applications, such as diagnosis, prognosis, drug screening, therapeutic development, and monitoring of disease progression.^[^ ^187^ ^]^ RGCs grown on a scaffold express specific neuronal biomarkers, indicating proper functionality.^[^ ^188^ ^]^ The combination of hiPSC‐derived cells and nanoscaffolds could serve as a therapeutic platform for personalized medicine and is potentially useful for neurophysiological analyses and transplantation for ophthalmic neuropathy treatment.^[^ ^189^ ^]^ hiPSCs may facilitate genetic disease modeling and the identification of novel drugs for treating retinal disorders.^[^ ^190^ ^]^ Further studies should explore whether, when combined with nanoscaffolds, hiPSCs can enhance stem cell function to treat retinal diseases.

## 5 Conclusion and Perspectives

### 5.1 Conclusion

Nanotechnology‐based therapeutics have shown significant potential to impact retinopathy outcomes in clinical settings, particularly by supporting retinal regeneration, as summarized in **Table** **1**. Our evaluation of studies conducted over the past 30 years indicates that nanotechnology can be fully applied to various aspects of retinal regeneration through delivery, support, and induction systems. However, further research is needed to develop nanomaterials with improved biocompatibility, superior physicochemical properties, and high retention. In particular, the utility and risks of nanoparticles require thorough validation.^[^ ^191^ ^]^ While most in vitro investigations have shown no adverse effects associated with nanoparticles,^[^ ^192^ ^]^ long‐term in vivo studies are crucial to understand the role of nanobiomaterials in regenerative medicine and to monitor potential side effects on other tissues. Additional pharmacokinetic, pharmacodynamic, and toxicological studies are necessary to improve our understanding of the bioeffects of nanobiomaterials on the retina. Hybrid nanostructures, such as coated nanoparticles, offer several advantages by overcoming the limitations of nanomaterials, thereby increasing their potential for use in biomedical applications. Furthermore, hybrid nanostructures can penetrate deeper into ocular tissues, enabling localized drug delivery at high concentrations, which enhances local drug efficacy.^[^ ^193^ ^]^ Although promising results have been reported, new sets of nanomaterial tools for retinal regeneration must be developed and optimized to meet future requirements for drug, gene, or substance delivery, as well as nanobiological engineering.

**Table 1:** Typical studies of nanomaterials used for retinal regeneration therapeutics.

Type	Disease	References	Nanostructure	Nanomaterial	Size range	Target tissue/cell	Delivery methods	Cytotoxicity
In vivo	Retinal and optic nerve disorders	[42]	NP	PEI‐NPs, GC‐NPs, HA‐NPs, HAS‐NPs, PEI/GC‐ heterogeneous‐NPs, HSA/GC‐heterogeneous‐NPs, and HSA/HA‐heterogeneous‐NPs.	210–340 nm	Retina	Intravitreal injection	No significant cytotoxicity
In vivo	AMD	[139]	NP	CeO_2_ NPs	NA	Retina	Intravitreal injection	Citations exclude cytotoxicity
In vivo	AMD	[138]	NP	CeO_2_ NPs	3–5 nm	Retina	Intravitreal injection	NA
In vivo & in vitro	Light‐induced degeneration of photoreceptor cells	[133]	NP	CeO_2_ NPs	NA	Retina	Intravitreal injection; Transfection	NA
In vivo	Light‐damage model disease	[136]	NP	CeO_2_ NPs	3–5 nm	Retina	Intravitreal Injection	No cytotoxicity
In vivo	LCA, Best's disease, some forms of RP and macular dystrophy	[163]	NP	Plasmid DNA CK30PEG10k NP	8–11 nm	RPE	Subretinal injection	No cytotoxicity
In vivo	LCA	[157]	NP	Plasmid DNA CK30PEG10k NP with S/MAR‐containing vector	NA	RPE	Subretinal injection	Citations exclude cytotoxicity
In vivo	RPE‐based diseases such as LCA	[201]	NP	Plasmid DNA CK30PEG10K NP	8–11 nm	RPE	Subretinal injection	Citations exclude cytotoxicity
In vivo	Stargardt disease	[162c]	NP	CK30PEG NP‐carrying DNA vectors	8–10 nm	Retina	Subretinal injection	Citations exclude cytotoxicity
In vivo	Chronic photoreceptor degenerative diseases, such as RP	[144]	NP	bFGF‐NP	585 nm	Retina	Intravitreal injection	Citations exclude cytotoxicity
In vivo	inherited retinal diseases such as RP and Stargardt's disease	[162b]	NP	Compacted DNA nanoparticles (DNA with CK30PEG10K)	<25 nm	RPE, choroid, and sclera (PECS) and Lens	Intravitreal injection	Citations exclude cytotoxicity
In vivo & in vitro	RD	[176]	MBVs	MBV with different concentration	NA	RGC	Intravitreal injection; Transfection	No cytotoxicity
In vivo	RP	[129, 130]	NP	Solid lipid nanoparticles loaded with myriocin	0.5 µm	Between photoreceptors and the retinal pigment epithelium	Eye drops	NA
In vivo	Chronic retinal disease	[139]	NP	CeO_2_ NPs	5–8 nm	Retina	Intravitreal injection	No cytotoxicity
In vivo & in vitro	XLRS	[152a]	NP	Dextran–protamine–DNA– SLN vector	200–270 nm	RPE	Intravitreal injection, subretinal injection and topical instillation; Transfection	No toxicity detected
In vitro	NA	[152b]	NP	Dextran–protamine–DNA– SLN vector	284 nm	Retina	Transfection	No cytotoxicity
In vivo	Blinding diseases	[154]	NP	Liposome‐protamine‐DNA complex	NA	RPE	Subretinal injection	NA
In vitro	AMD	[155]	Copolymers	PLLA with PLGA	20–70 mm	RPE	Treated	NA
In vivo	RP	[157]	NPs	Compacted DNA NPs	8 nm	RPE	Subretinal injection	Citations exclude cytotoxicity
in vitro	Retinal degeneration disease	[175]	Nanofibers	Core‐shell fibrous scaffold from PEG/PCL	NA	RPE	Treated	NA
In vivo	AMD, RP and Retinal dystrophy	[177]	NPs	P3HT	304 nm	RPE	Subretinal injection	NA
In vivo & in vitro	RP and AMD	[182]	Nanofibers	PCL	5 mm	Retinal progenitor cells	Subretinal injection	NA

Abbreviation: NP, Nanoparticles; PEI, Polyethyleneimine; GC, Glycol Chitosan; HA, Hyaluronic Acid; HAS, Human Serum Albumin; ARPE‐19, Adult Retinal Pigment Epithelial cell line‐19; PLGA, Poly(lactic‐co‐glycolic acid); CeO_2_, Cerium Oxide; VEGF, Vascular Endothelial Growth Factor; Vldlr, Very Low Density Lipoprotein Receptor; AMD, Age‐related Macular Degeneration; LCA, Leber's Congenital Amaurosis; RP, Retinitis Pigmentosa; RD, Retinal Detachment; XLRS, X‐linked juvenile retinoschisis; PLLA, poly(L‐lactic acid); PLGA, poly(D,L‐lactic‐glycolic acid); PEG, Poly Ethylene Glycol; PCL, Poly Caprolactone; P3HT, poly[3‐hexylthiophene]; NA, Not Available.

### 5.2 Perspectives

Despite the progress made in utilizing nanostructures in biomedicine, systematic research is essential to overcome various challenges. The primary cause of irreversible eye damage stems from a range of ophthalmic diseases. While in vitro studies have indicated minimal toxic effects of nanoparticles and nanowires, comprehensive in vivo studies are necessary to fully discern the significance of these nanobiomaterials in regenerative medicine. Currently, the application of nanobiomaterials in eye treatments has been effective in preventing and treating various forms of retinopathy. However, their administration through invasive methods may lead to inflammation, infection, or retinal detachment, potentially resulting in vision loss. These drawbacks not only affect patients’ quality of life but also pose significant economic challenges for the health care system. Consequently, additional pharmacokinetic, pharmacodynamic, and toxicological studies are needed to fully understand the biological effects of nanobiomaterials on the retina. Advancements in nanobiomaterial engineering could pave the way for novel retinal therapeutic and diagnostic procedures. The use of noble metals, bioinspired magnetic nanoparticles, and nanowires could enhance real‐time imaging, thereby addressing the shortcomings of conventional treatments. Hybrid nanostructures play a critical role by combining the advantages and mitigating the disadvantages of nanomaterials. These structures can penetrate deeper into ocular regions, allowing for localized, high‐dose drug delivery and increasing local drug concentrations. Recent studies have suggested that nanoparticle‐based eye‐drop formulations can effectively deliver specific drugs to the retina in rats. Therefore, improving the noninvasive delivery of nanomaterials across the blood‒retinal barrier could lead to significant progress in biomedicine. One potential strategy is to combine stem cells with hybrid nanostructures to stimulate retinal formation and regeneration. Such an approach could address the inconsistencies between in vitro and in vivo studies and manage neuronal signals, translating them into the extracellular matrix and subsequently transmitting them to the brain to generate clear vision. Therefore, the combination of hybrid nanostructures and stem cells represents a promising, bottom‐up approach for future research into retinal rejuvenation. Since stem cells have the ability to proliferate and are susceptibility to genetic mutations, therapeutic techniques using exogenous stem cells have the risk of inducing tumors, which limits their clinical application. Nevertheless, the controversy surrounding stem cell therapies continues to provide a degree of valuable exploration and insight for the development of novel therapeutic modalities.

## Conflict of Interest

The authors declare no conflict of interest.

## Author Contributions

C.Y., L.D., and Y.L. contributed equally to this work. C.Y. and L.D. helped writing the manuscript. Y.L. designed the structure of the manuscript and wrote the manuscript. X.S., R.Z., W.Z., H.W., H.L., and Y.L. help collect the information. Z.L., D.L., and W.W. revised the manuscript.
